# Digital Health Intervention on Awareness of Vaccination Against Influenza Among Adults With Diabetes: Pragmatic Randomized Follow-Up Study

**DOI:** 10.2196/68936

**Published:** 2025-04-10

**Authors:** Yifat Fundoiano-Hershcovitz, Felix Lee, Catherine Stanger, Inbar Breuer Asher, David L Horwitz, Omar Manejwala, Jan Liska, David Kerr

**Affiliations:** 1 Dario Health Caesarea Israel; 2 Sanofi Bridgewater, NJ United States; 3 Geisel School of Medicine Center for Technology and Behavioral Health Hanover, NH United States; 4 DLH Biomedical Consulting Las Vegas, NV United States; 5 Sanofi (France) Paris France; 6 Center for Health Systems Research, Sutter Health Santa Barbara United States

**Keywords:** digital health, diabetes management, influenza vaccination, flu vaccination awareness, mobile health

## Abstract

**Background:**

Diabetes mellitus significantly increases the risk of severe complications from influenza, necessitating targeted vaccination efforts. Despite vaccination being the most effective preventive measure, coverage remains below the World Health Organization’s targets, partly due to limited awareness among patients. This study evaluated a digital health intervention aimed at improving influenza vaccination rates among adults with diabetes.

**Objective:**

This study aimed to demonstrate the effectiveness of digital health platforms in increasing vaccination rates among people with diabetes and to emphasize the impact of tailored messaging frequency on patient engagement and health behavior change. We hypothesized that digital tools providing empirical evidence of increased health risk awareness can effectively drive preventive actions.

**Methods:**

The study leveraged the Dario (Dario Health Corp) digital health platform to retrospectively analyze data from 64,904 users with diabetes assigned by the platform into three groups: (1) Group A received previously studied monthly flu nudge messages; (2) Group B received an adapted intervention with 2-3 monthly messages; (3) Group C served as the control with no intervention. Surveys were conducted at baseline, 3 months, and 6 months to assess vaccination status, awareness of influenza risks, and recollection of educational content. Statistical analyses, including logistic regression, chi-square tests, and *t* tests, were used to evaluate differences between groups.

**Results:**

Out of 64,904 users, 8431 completed the surveys. Vaccination rates were 71.0% in group A, 71.9% in group B, and 70.5% in group C. Group B showed significantly higher awareness of influenza risks compared with the control group odds ratio (OR; OR 1.35, 95% CI 1.12-1.63; *P*=.001), while group A did not (OR 1.10, 95% CI 0.92-1.32; *P*=.27). Recollection of educational content was also higher in groups A (OR 1.29, 95% CI 1.07-1.56; *P*=.008) and B (OR 1.92, 95% CI 1.59-2.33; *P*<.001) compared with the control. In addition, a significant correlation between awareness and vaccination rates was found only in group B (*χ*^2^(df=1)=6.12, *P*=.01).

**Conclusions:**

The adapted digital intervention (group B) effectively increased awareness of influenza risks and recollection of educational content, which correlated with the higher trend in vaccination rates. This study demonstrates the potential of digital health tools to enhance influenza vaccination among people with diabetes by improving risk awareness and education. Further research should focus on optimizing these interventions to achieve significant improvements in vaccination uptake and overall public health outcomes.

**Trial Registration:**

ClinicalTrials.gov NCT06840236; https://clinicaltrials.gov/study/NCT06840236

## Introduction

Diabetes mellitus is one of the most common chronic diseases [1], contributing significantly to the global health care burden [2] due to the risk of developing serious complications including cardiovascular disease, kidney failure, blindness, and lower limb amputation [3]. In addition, people with diabetes also have an increased risk of developing serious medical complications from influenza [4] including uncontrolled diabetes leading to unscheduled attendance at the emergency room or hospital admission, pneumonia, premature death, and acute cardiovascular complications [5]. As a consequence, the World Health Organization (WHO) considers individuals with diabetes to be a high-risk group with a greater susceptibility for developing more severe and complicated influenza viral infections [6].

In addition, many patients with diabetes have other conditions that can increase the severity of influenza. As an example, approximately 90% of patients living with type 2 diabetes are overweight, and obesity is an independent risk factor for severe influenza infection [4].

Age is cited as another risk factor by the Centers for Disease Control and Prevention (CDC); specifically, people aged 65 years and older are at a higher risk of developing serious influenza-associated complications [7] with up to 90% of excess deaths occurring in this age group [8]. This may be because, with increasing age, the innate and adaptive immune responses gradually deteriorate, manifesting in a reduced capacity to respond to infection and immunization [9]. Therefore, older adults need to take optimal measures to prevent infectious diseases.

Vaccination remains the most effective primary prevention method against influenza [5] and is considered responsible for significantly lowering mortality and reducing care costs and hospitalization [10]. Especially for those who are at risk from influenza complications, vaccination is highly recommended on an annual basis [11], including people with diabetes [7]. The WHO has set targets for influenza vaccination rates, is aiming for 70% coverage in the general population [5] and 75% in high-risk groups [12]. However, as reported by the CDC for the 2022-23 period, vaccination rates in the United States fell short of these goals, with overall coverage at 46.9% among adults [13]. Both reported rates were 2.5 and 4.2 percentage points lower compared with coverage rates during the 2021-2022 season, respectively. Moreover, among adults aged ≥18 years with diabetes for the period of August 2007 to August 2017, the vaccination coverage ranged from 62.6%-64.8% [14].

Limited awareness is one of the barriers to vaccination against influenza as evidenced by the fact that patients who had consulted their general practitioner about vaccination were more likely to receive their seasonal influenza vaccine regularly over the subsequent 5 years [15]. These findings align with previous research, indicating that recommendations from medical professionals significantly boost vaccination coverage [16]. A lack of understanding regarding influenza-related risks and low awareness of the benefits of vaccination for people with diabetes may influence vaccination behavior. Other barriers reported were negative attitudes toward health care, direct and indirect costs, Preference to receive the COVID-19 pandemic vaccine over the influenza vaccine or impact of previous vaccination experience on future uptake [17].

Many strategies have been deployed to promote vaccinations and boost coverage: advising text messages, reminders, telephone outreach, and brief educational interventions. However, only a subset of these strategies has undergone evaluation [18]. Using digital messaging as an approach is a novel and cost-effective solution to directly address the lack of knowledge and education about influenza, as well as to overcome barriers to vaccination for people with diabetes. To achieve effective behavior change, the theoretical model COM-B (Capability, Opportunity, Motivation, and Behavior) proposes 3 factors that interact to influence behavior [19]. The use of the COM-B model in this context is particularly effective for addressing the limited awareness of the risks associated with not getting vaccinated and for guiding strategies aimed at behavior change. Capability refers to the ability to engage in processes (both psychological and physical) necessary to perform the wanted behavior, the intervention can address this by using educational messages that enhance knowledge and understanding, helping individuals recognize the personal and public health benefits of vaccination. Opportunity refers to environmental factors (both social and physical) that influence behavior, in the intervention, providing a CDC Flu Finder Widget ensures physical opportunity by making it easier for users to locate nearby vaccination clinics. Motivation refers to beliefs and emotions or impulses that direct behavior, by emphasizing the benefits of vaccination and aligning messaging with personal health goals (eg, reducing the risk of severe illness), the intervention taps into both reflective and automatic forms of motivation. Applying COM-B to the early stages of intervention development can help to identify intervention components that address potential obstacles in order for the behavior change to occur [19,20]. COM-B ensures that the intervention is holistic, targeting multiple dimensions simultaneously.

By using the COM-B approach for a digital health intervention, there is potential to enhance individuals’ psychological and physical capacity through educational messages, thereby increasing their capability for behavior change. Using a digital messaging approach in a “nudge” tool may therefore be effective at increasing vaccination rates by creating the opportunity as a factor that lies outside the individual [21]. “Nudge” tool design primarily seeks to modify the external environment (eg, timing and frequency of reminders and the framing of the message) to ensure the information reaches individuals at the right moment. Digital health can be used for a combination of setting goals; self-care behaviors and feedback on behavior creates dynamic personalization that enhances motivation for behavior change [22] and has the advantage of being able to provide medical care services anytime and anywhere without being restricted by geographic location and time. During the COVID-19 pandemic approximately 33%-52% of people with diabetes used mobile apps to manage their health [23].

The potential for low-cost psychological interventions to change behavior has been documented in previous research [24]. In the previously published randomized trial of digital intervention to increase influenza vaccination rates in people with diabetes, the vaccination rate was 3.1% higher in the intervention group than the control group [5]. Given the increased burden of influenza for people with diabetes, even small improvements in vaccination rates could substantially reduce the number of patients having severe complications.

Implementing evidence from clinical research trials into the real-world can be challenging [25,26]. We adopted a pragmatic approach to evaluate the effectiveness of an adapted digital nudge intervention that had demonstrated positive results in a previous randomized controlled trial (RCT) to increase influenza vaccination rates for people with diabetes in real-world setting. The primary objective was to examine the difference in self-reported influenza vaccination rates in three groups: (1) people with diabetes who received the intervention using a previously published approach (group A), (2) people with diabetes who received an adapted digital intervention (group B) and (3) people with diabetes who received no intervention (group C or control). A secondary aim was to investigate whether the intervention led to differences in awareness and knowledge across study groups and examined the relationship between awareness of influenza risks and vaccination rates.

## Methods

### Study Population

This study included a cohort of subjects from the United States using a digital health tool (Dario [27]) who reported in the smartphone app during registration that they had a diagnosis of type 1 or 2 diabetes and who were active on the platform (logged into the app) during the 12-month period up to September 2022.

### Study Design

This analysis examined data collected between September 2022 and March 2023 to evaluate the effectiveness of an adapted digital nudge intervention designed to increase influenza vaccination rates among people with diabetes in a real-world setting. The intervention builds on a previously conducted RCT [5]. Users in that trial were automatically assigned to 3 groups via the customer engagement platform using a randomization algorithm to assign each selected user to one of the test variations. This ensured that each user had an equal chance of being placed in any of the groups. The 3 groups are defined as follows: group A received flu nudge messages as described previously in a randomized clinical trial [5] with one message given each month for 6 months; Group B received similar messages that were adapted in frequency with 2-3 messages per month for 6 months and matched the Dario platform’s user experience (Adapted Intervention below); group C received no flu nudges (control). No compensation was offered.

**Figure 1:**
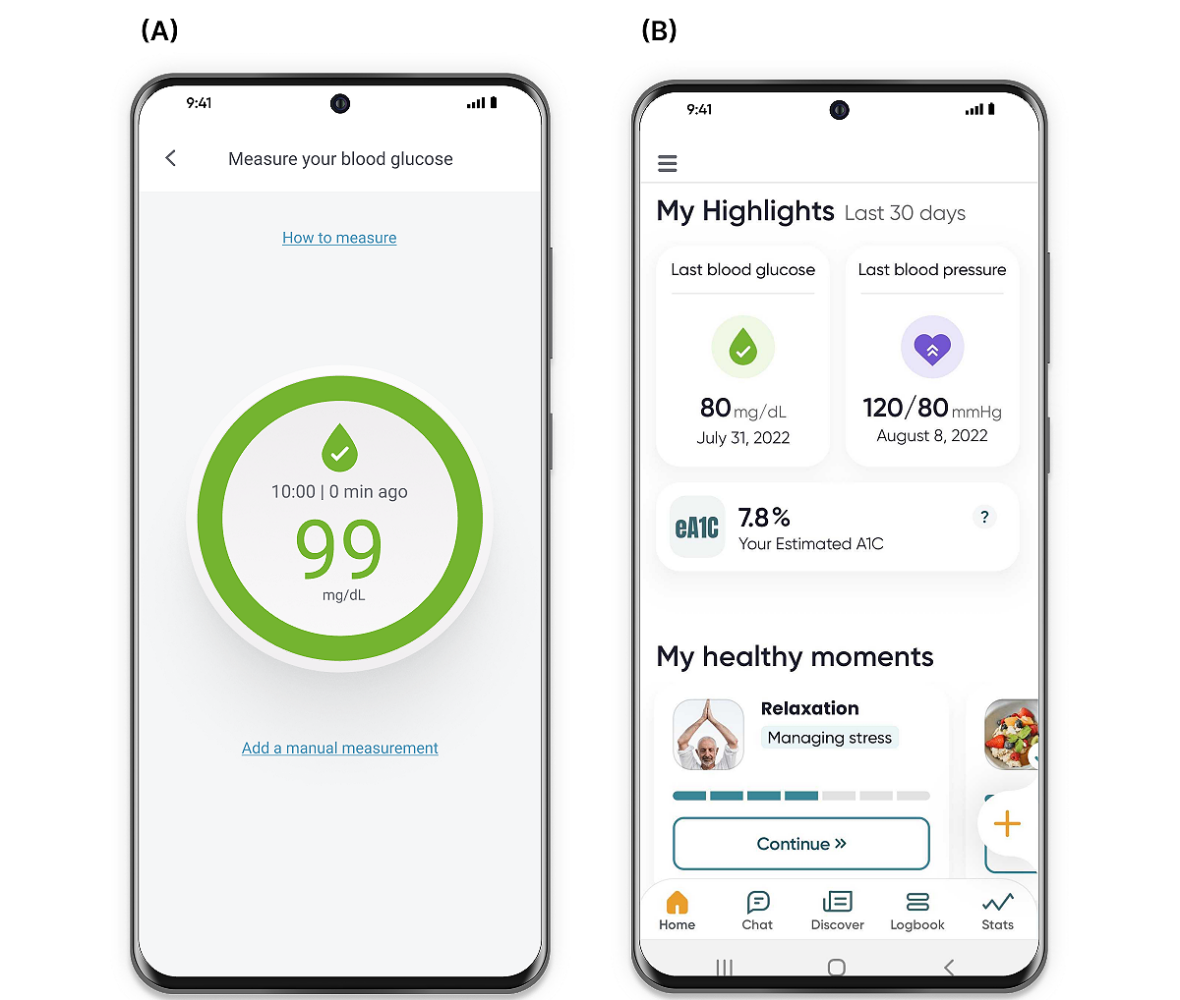
Dario mobile app platform. (A) Measurement screen allows the displaying of the blood glucose measurement. (B) Main screen presenting a summary of measurements and activities.

**Figure 2:**
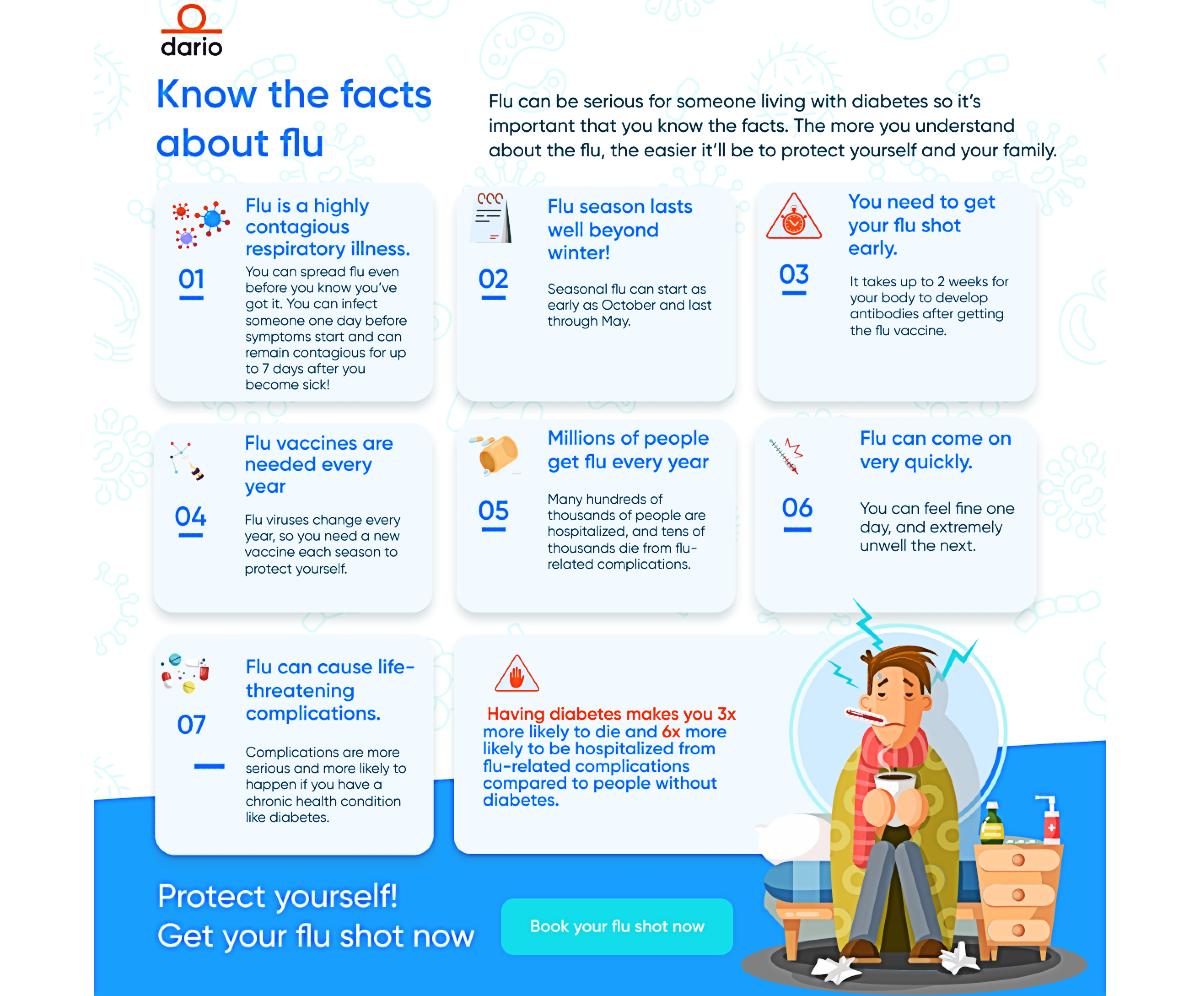
An example of an educational content delivered in a digital message to members-the topic was “facts about flu.”.

Group A received 6 monthly messages as structured in the previous RCT. The intervention messages included education and recommendations, designed and delivered similarly to the previously published data [5]. Each of the messages delivered to Group B were structured in two parts: (1) educational content, and (2) a call to action for the user to complete. Educational messages were based upon data from the US CDC, Vaccines.gov, and the American Diabetes Association [28-30]. Messages were communicated via the Dario app using topics such as facts about influenza and vaccine benefits for people with diabetes, for example occurring around World Diabetes Day and Thanksgiving in November. Calls to action encouraged the users to complete specified actions such as locating the nearest clinic offering flu shots (CDC Flu Finder Widget) or planning prompts (Figures 1 and 2).

### Platform and Intervention Content

This study used the Dario multicondition digital therapeutics platform for chronic conditions management including diabetes, hypertension, and obesity. The platform combines a glucometer with a smartphone app that is available for both Android and iOS devices. The blood glucose monitoring system consists of a small pocket-sized holder for strips, a lancet, and the meter. The meter is removed from the holder and plugged directly into the smartphone, effectively converting the smartphone into the display screen for the meter.

The COM-B model provided an evidence-based approach to guide the intervention ensures that it addresses the multifaceted barriers to vaccination behavior, making it more likely to achieve meaningful and lasting behavior change. Limited awareness about the risks of not being vaccinated indicates a gap in psychological capability, knowledge, and understanding of why the vaccine is critical. The intervention can address this by using educational messages that enhance knowledge and understanding, helping individuals recognize the personal and public health benefits of vaccination. In the intervention, providing a CDC Flu Finder Widget ensures physical opportunity by making it easier for users to locate nearby vaccination clinics. Emphasizing the benefits of vaccination and aligning messaging with personal health goals (eg, reducing the risk of severe illness) and planning prompts enhance motivation by making the behavior easier to initiate.

Educational messages were developed using data from the US CDC, Vaccines.gov, and the American Diabetes Association [28,29,31]. Examples of topics covered in the intervention include (1) winter is coming; (2) flu can be very serious; (3) facts about flu; (4) vaccines benefits; (5) prepare yourself for vaccination; (6) myths and facts about flu for 65 and above; (7) vaccines and diabetes; (8) diabetes day; Happy Thanksgiving; (9) flu versus Covid; (10) symptoms and treatment; and (11) seek emergency medical care. An example of a message is provided in Textbox 1:

**Example of an educational message.**

**“Winter is Coming–and So is the Flu”**

Flu season is just around the corner and many experts are predicting that this season could be worse than before the COVID pandemic.

The flu is a highly contagious respiratory illness. Anyone can get the flu but for people with a chronic health condition like diabetes, it can cause serious illness and even death.

**Diabetes Puts You at an Increased Risk of Flu and Serious Flu-Related Complications**

This, combined with the ongoing COVID-19 pandemic, is why you must protect yourself, and protect yourself early!

**CDC Recommends Annual Flu Vaccination to be the Best Way to Protect Yourself Against Flu.**

Prepare yourself for the winter flu season

“Book your flu shot now”

### Measures

All users were sent an online baseline survey before any intervention content, a midstudy assessment at 3 months, and a final assessment at 6 months (Multimedia Appendix 1). Completion of midstudy and final assessment was not predicated on completion of the baseline assessment. The endpoint of influenza vaccination status was collected in the 3- or 6-month surveys (Multimedia Appendix 2). Questions on demographics, and influenza vaccination status were asked of all members who participated (groups A, B, C) while questions on content recollection and perceptions of the interventions were asked of participants who received interventions (groups A and B). The digital intervention flow was applied the same way as in the previous RCT [5].

### Statistical Analysis

#### Baseline Characteristics Analysis

A demographic descriptive analysis providing absolute frequencies and percentages of users who completed 3-month and 6-month surveys (completers) and of users who did not complete the surveys (noncompleters) was performed. For the comparison of demographic variables, an independent sample *t* test was used and for the comparison of frequencies, comparisons were made using the chi-square test. Variables were compared at the 5% significance level using 2-sided tests or 2-sided 95% CI unless otherwise specified. Cramer V test was applied for measuring the effect size of correlation between categorical fields and Cohen *d* for measuring the effect size of the differences between 2 group means [32].

#### Differences in Vaccination Rates and in Content Recollection

A logistic regression model was used to estimate the probability of independent correlations between survey answers and the study groups. The *P* values, odds ratios (ORs), and 95% CIs associated with each of the β parameter estimates were reported.

### Ethical Considerations

All data used for the analysis were anonymized before extraction for this study. The survey received a determination document reflecting that no formal institutional review board review is required from the institutional review board under the Ethical and Independent Review Services, a professional review board, which issued the institutional review board exemption for this study (ID# 22166) [33]. The users who participated in the study were provided with a Terms of Use document stating the legally valid consent of the end user for the company to collect and access their information. The use of the app, site, or services shall be deemed to constitute user consent to be legally bound by the terms of use and the privacy policy [31].

## Results

### Baseline Characteristics of Study Population

**Figure 3:**
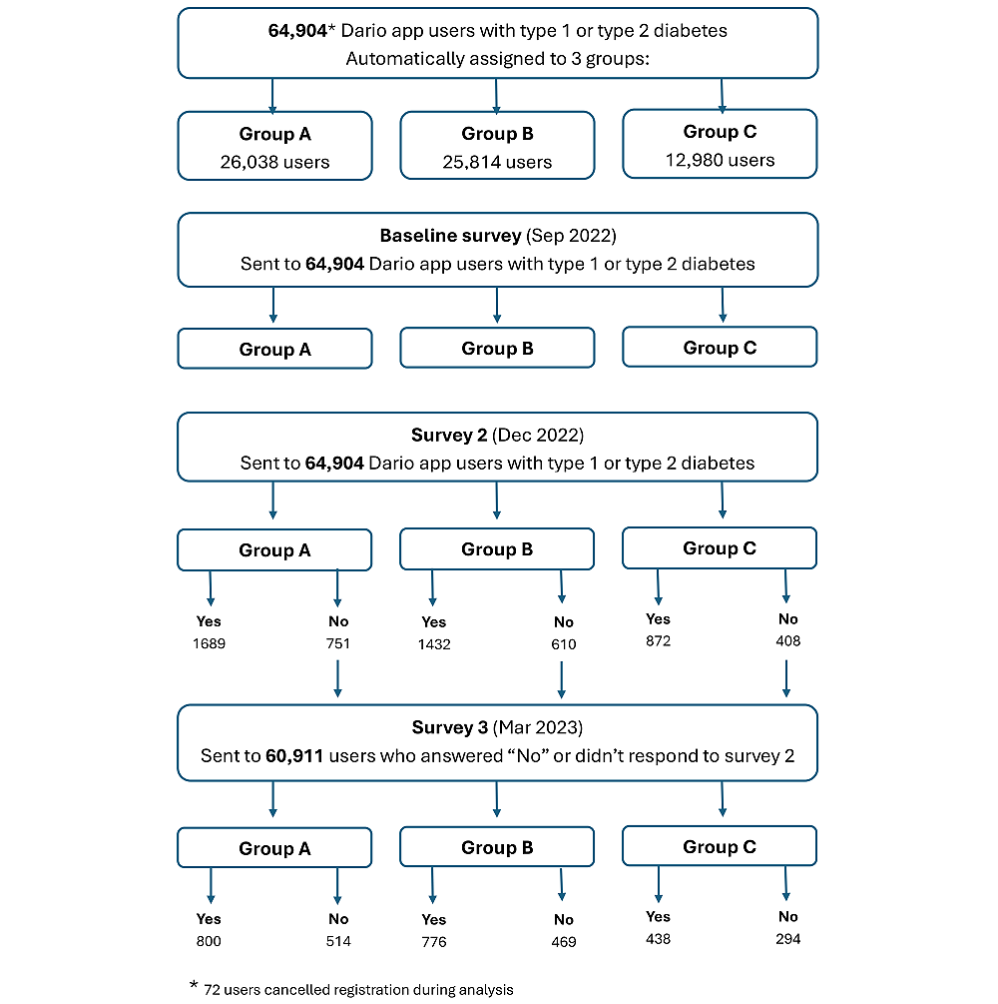
Study population. Flow chart showing the definition of the study population including inclusion criteria and surveys completion cohorts.

A sample of 64,904 users meeting the criteria described above was automatically assigned to the 3 random groups. The mean age of study participants was 57.5 (SD 13.2) and 52% were male. Out of 64,904 users, a total of 8431 users with diabetes completed influenza vaccination status at surveys (2) or (3) and were defined as completers-group A (n=3505), group B (n=3068), and group C (n=1858; Figure 3). A total of 56,473 users did not complete surveys (2) or (3) and were defined as noncompleters. A comparison of the demographic variables between completers and noncompleters is shown in Table 1.

**Table 1:** Participants demographics for Dario users who completed vaccination status in surveys (2) or (3) (completers) and those who did not complete either of the 2 surveys (noncompleters).

			Completers (n=8431)		Noncompleters (n=56,473)		*P* value			Effec*t* size	
Age (years), mean (SD)			63.7 (11)		56.6 (13.2)		<.001^a^			Cohen *d*=0.58	
**Gender, n (%)**								<.001^b^			Cramer V=0.03
	Female	4376 (52.1)		26728 (47.5)							
	Male	5022 (47.9)		29508 (52.5)							
	Other	0 (0)		5 (0)							
BMI, mean (SD)			33.2 (7.8)		33.4 (8.4)		<.05^a^			Cohen *d*=0.02	
Year of diagnosis (duration of diabetes), mean (SD)			2009.7 (50.7)		2012.2 (50.8)		<.001^a^			Cohen *d*=0.05	
**Diabetes type, n (%)**								<.001^b^			Cramer V=0.06
	Type 1	453 (5.4)		5955 (10.6)							
	Type 2	7933 (94.1)		50272 (89.1)							
	Other	32 (0.4)		156 (0.3)							
**Insulin treatment, n (%)**								<.001^b^			Cramer V=0.01
	No	5730 (69.2)		36583 (69.2)							
	Pen	2248 (27.6)		14716 (27.8)							
	Pump	169 (2.1)		1592 (3)							
**Comorbidities, n (%)**								<.001^b^			Cramer V=0.13
	Cardio-metabolic^c^	3930 (56.6)		25211 (59)							
	MSK^d^	587 (8.5)		1487 (3.5)							
	BH^e^	458 (6.6)		6583 (15.4)							
	None	1966 (28.3)		9443 (22.1)							

^a^Two sample *t* test for comparing mean samples.

^b^Chi-square test of independence.

^c^Cardio-metabolic (cardiovascular, cerebrovascular, endocrine, high blood lipids, hypertension, kidney disease, obesity, and vascular).

^d^MSK: musculoskeletal.

^e^BH: behavioral health.

Based on the chi-square and *t*-test outcomes, survey completers and noncompleters were significantly different in their demographics. However, the numerical difference was minimal, as indicated by Cramer V and Cohen *d* tests. Cramer’s V and Cohen *d* test outcomes reflected a small effect for gender, BMI, insulin treatment, diabetes type, year of diagnosis, and comorbidities. A large effect was observed for age (Cohen *d*=.58), with completers being significantly older than noncompleters (*P*<.001). Overall, older adults (>65 years) showed higher intent in pursuing influenza vaccination, similar to other reports [34].

The influenza vaccination intent in all 3 study groups was indicated by the response to the baseline survey 1 question, “Do you plan to get a flu shot this flu season?” A chi-square test was used to show differences in frequencies between the groups. The results of the test demonstrated that there were no statistically significant differences between the groups in their intent to get vaccinated (*χ*^2^(df=4)=4.98, *P*=.29) as presented in Table 2. A total number of 6059 users responded to the question “Do you plan to get a flu shot this flu season?” 2425 are from group A, 2422 from group B, and 1212 from group C (Table 2).

**Table 2:** Baseline intent to get influenza vaccination. Group A: published randomized controlled trial–based intervention; group B: Dario-adapted intervention; group C: control. Data collection of members’ responses to the question, “Do you plan to get a flu shot this flu season?” in survey 1.

Answer	Group				Total n, (%)
	A, n (%)	B, n (%)	C, n (%)		
Yes	1822 (75.1)	1796 (74.2)	878 (72.4)	4496 (74.2)	
No	432 (17.8)	456 (18.8)	253 (20.9)	1141 (18.8)	
Have not decided yet	171 (7.1)	170 (7)	81 (6.7)	422 (7)	
Total	2425 (100)	2422 (100)	1212 (100)	6059 (100)	

Chi-square test of independence was performed to examine the relationship between the study groups and their response to the question, “Do you plan to get a flu shot this flu season.” The results indicated a nonsignificant association between the 2 variables (*χ*^2^(df=4)=4.98, *P*=.29). All expected cell frequencies were greater than 5, satisfying the assumption for the chi-square test.

### Differences in Vaccination Rates Between the Study Groups

A total of 8431 users with diabetes self-reported influenza vaccination status in surveys (2) or (3). After 6 months, ratios of 71.0% (2489/3505) of group A reported getting vaccinated versus 71.9% (2207/3068) in group B and 70.5% (1310/1858) in group C (*χ*^2^(df=2)=1.30, *P*=.52) as presented in Table 3.

Chi-square test of independence was performed to examine the relationship between the study groups and their reported vaccination status. The results indicated a nonsignificant association between the 2 variables (*χ*^2^(df=2)=1.30, *P*=.52). All expected cell frequencies were greater than 5, satisfying the assumption for the chi-square test.

**Table 3:** Vaccination rate per completed intervention for people with diabetes. The results were collected from people who completed vaccination status at survey 2 or 3.

Answer	Group				Total, n (%)
	A, n (%)	B, n (%)	C, n (%)		
Yes	2489 (71)	2207 (71.9)	1310 (70.5)	6006 (71.2)	
No	1016 (29)	861 (28.1)	548 (29.5)	2425 (28.8)	
Total	3505 (100)	3068 (100)	1858 (100)	8431 (100)	

### Content Related Differences Between the Groups

#### First Analysis: Differences in Awareness and Knowledge Retention Levels Between the Groups

Based on the significant association revealed by the chi-square test between the study groups and their response to the question, “Do you know the risks getting the flu has for someone with diabetes?” (*χ*^2^(df=2)=11.27, *P*=.04), we proceeded with a logistic regression analysis to compare the groups to the control. A logistic regression analysis was applied to evaluate the differences in awareness and knowledge rates assessed in survey 2 between the study groups, reflected by the response to the question, “Do you know the risks getting the flu has for someone with diabetes?” A ratio of 81.7% (1428/1747) of group B reported “Yes” versus 78.5% (1621/2065) of group A and 76.8% (813/1059) of group C. In comparison to group C (control), group B exhibited 1.35 times greater awareness of the risks associated with influenza compared to the control group (OR 1.35, 95% CI 1.12-1.63; *P*=.001). However, group A did not demonstrate statistically significant differences in risk awareness compared with the control group (OR 1.10, 95% CI 0.92-1.32; *P*=.27; Table 4).

**Table 4:** Awareness and knowledge retention rate per completed intervention for people with diabetes. The results were collected in survey 2 only from people who completed the following question: “Do you know the risks getting the flu has for someone with diabetes?”

Answer	Group				Total, n (%)
	A, n (%)	B, n (%)	C, n (%)		
Yes	1621 (78.5)	1428 (81.7)	813 (76.8)	3862 (79.3)	
No	444 (21.5)	319 (18.3)	246 (23.2)	1009 (20.7)	
Total	2065 (100)	1747 (100)	1059 (100)	4871 (100)	

Chi-square test of independence was performed to examine the relationship between the study groups and their response to the question, “Do you know the risks getting the flu has for someone with diabetes?” The results indicated a significant association between the 2 variables (*χ*^2^(df=2)=11.27, *P*=.04). All expected cell frequencies were greater than 5, satisfying the assumption for the chi-square test.

Furthermore, the relationship between the awareness of the risk from influenza and vaccination rate only in group B using chi-square test was tested. There were significant differences in vaccination frequencies between individuals who reported awareness of the risks to those who were not aware (*χ*^2^(df=1)=6.12, *P*=.01). Table 5 demonstrates the frequencies between the groups, showing a higher percentage of vaccinated users who reported awareness of the influenza risks than vaccinated users who were not aware (81.5% and 75.2%, respectively).

**Table 5:** The relationship between awareness of influenza risks for persons with diabetes and vaccination rate in group B.

“Do you know the risks getting the flu has for someone with diabetes?”	“Did you get vaccinated?”			Total, n (%)
	Yes, n (%)	No, n (%)		
Yes	1164 (81.5)	264 (18.5)	1428 (100)	
No	240 (75.2)	79 (24.8)	319 (100)	
Total	1404 (80.4)	343 (19.6)	1747 (100)	

Chi-square test of independence was performed to examine the relationship between the members reported vaccination status and their response to the question, “Do you know the risks getting the flu has for someone with diabetes?” The results indicated a significant association between the two variables (*χ*^2^(df=1)=6.12, *P*=.01). All expected cell frequencies were greater than 5, satisfying the assumption for the chi-square test.

#### Second Analysis: Differences in Recollection of Educational Content Between the Groups

Based on the significant association revealed by the chi-square test between the study groups and their response to the question, “Do you remember seeing any of these messages?” (*χ*^2^(df=2)=50.41, *P*<.001), we proceeded with an additional logistic regression analysis to compare the groups to the control. The logistic regression analysis was applied to investigate the differences in the probabilities of educational content recollection between the study groups reflected by the response to the question, “Do you remember seeing any of these messages?” in survey 3. Among them, 56.6% (666/1176) of group B reported “Yes” compared to 46.7% (575/1231) in group A and 40.4% (281/695) in group C. Groups A and B exhibited 1.29 times (OR 1.29, 95% CI 1.07-1.56; *P*=.008) and 1.92 times (OR 1.92, 95% CI 1.59-2.33) greater recollection of educational content compared to the control group, respectively (*P*<.001; Table 6).

Chi-square test of independence was performed to examine the relationship between the study groups and their response to the question, “Do you remember seeing any of these messages?” The results indicated a significant association between the 2 variables (*χ*^2^(df=2)=50.41, *P*<.001). All expected cell frequencies were greater than 5, satisfying the assumption for the chi-square test.

**Table 6:** Recollection of educational content per completed intervention for people with diabetes. The results were collected in survey 3 only from people who completed the following question: “Do you remember seeing any of these messages?”

Answer	Group			Total, n (%)
	A, n (%)	B, n (%)	C, n (%)	
Yes	575 (46.7)	666 (56.6)	281 (40.4)	1522 (49.1)
No	656 (53.3)	510 (43.4)	414 (59.6)	1580 (50.9)
Total	1231 (100)	1176 (100)	695 (100)	3102 (100)

## Discussion

### Principal Findings

This study used an adapted digital intervention within a diabetes digital therapeutic platform to enhance influenza vaccination rates for people with diabetes. Vaccination rates of the groups were 71.9% in group B who received the adapted digital intervention compared with the rates in group A with a previously published digital intervention and group C as control with 71.0% and 70.5%, respectively. Expanding on that, in group A, there was a higher intention to get vaccinated at baseline compared to group B (75.1% vs 74.2%), but a lower vaccination rate was reported at either survey 2 or 3 (71.0% vs 71.9%). Conversely, in group B, there was a lower intention compared to group A, yet a higher vaccination rate was observed although it was not statistically significant. Greater awareness of the risks associated with influenza in diabetes was demonstrated in group B, up to 1.35 times more than the control group, while group A did not show a difference in the awareness to risk compared to the control. Furthermore, a significant correlation between risk awareness and vaccination rates was remarked only in group B indicating how awareness of influenza risks, provided through the digital journey, relates to higher vaccination rates among people with diabetes. The recollection of educational materials was significantly higher in group B, with a 1.92-fold increase compared to the control group. Group A also exhibited a higher recollection of educational materials, with a 1.29-fold increase compared to the control group.

To our knowledge, this study was one of the first to demonstrating the use a digital health intervention to promote influenza vaccination for people with diabetes in a real-world setting. Previous randomized studies of digital health interventions demonstrated rates of influenza vaccination with an absolute difference of 2.06% or 3.1% differences versus the control group [5,35], while in this study the difference is 1.4% versus the control group. Compared with previous studies, where the average baseline of vaccination rates was around 60% [5,35], our control group in this study showed rates of 70.5%. This presents a significant challenge to improve upon. It indicates that our initial cohort was already highly motivated in their health, which may have made it more challenging to achieve further improvement. Moreover, previous studies reported that there was compensation provided as a motivating factor for vaccination report and for completion of the interventions [5,35].

This study showed a vaccination rate trend of 1.4% compared with the control group. The current reported prevalence of diabetes is around 38.4 million Americans [36]. Increasing vaccination rates by 1.4% could result in approximately 532,000 people with diabetes getting immunized, and potentially avoiding complications associated with diabetes [35,37]. Ultimately, an increase of approximately 2% in vaccination rate would likely translate to substantial reductions in morbidity, mortality, and costs to the health care system, as well as potential improvements in the quality of life if applied at scale [12,35,37]. The US National Committee for Quality Assurance (NCQA) supports the broad use of the Health Care Effectiveness Data and Information Set measure specifications to evaluate and drive health care quality [38]. NCQA recommends flu vaccine for all adults and reports that vaccinations can reduce flu-related hospitalizations by 71% [39]. This reduction in health care resource utilization is even more pronounced in people with diabetes, with flu vaccination having been associated with reduced hospitalizations by 79% [40]. Findings on the cost-effectiveness of digital interventions show a growing body of evidence suggesting generally favorable effects on costs and health outcomes [41]. Specifically for flu, it costs an estimated US $11.2 billion annually in direct and indirect costs [42]. Flu vaccination is the most effective way to protect employees from becoming sick from the virus as stated by NCQA in the published report “ACT ON THE FACTS-Flu Immunizations” [43,44]. The reasons for increased susceptibility of people with diabetes to influenza-associated complications remains unclear. An impaired immune response has been hypothesized as responsible for an increased risk of infection as well as the complications that are accompanied by it, yet the evidence is inconclusive [7]. Alternatively, hyperglycemia may increase the risk and severity of bacterial infections secondary to influenza infection [4]. Hyperglycemia can reduce immune cell recruitment, neutrophil degranulation [8], impair complement activation [9], and immune cell phagocytosis, which together can inhibit the immune response against influenza virus infection [4,6,10].

The correlation between awareness of influenza risks in the population of people living with diabetes and actual vaccination rates highlights the potential of a digital platform to promote and effectively drive behavioral change. The benefit of a multi condition digital health platform lies in its ability to deliver evidence-based interventions tailored to individual patient needs. Implementing changes requires the design of effective and efficient behavioral interventions. The influence of digital health on users’ behavior change in this study may be explained by the COM-B model. COM-B model conceptualizes behavior as a part of a system of interacting factors [45] and has been effectively applied to many health behaviors [46]. The COM-B model identifies (capability, opportunity, and motivation) as crucial factors influencing behavior change. It is widely applied in health care settings and for interventions targeting lifestyle changes such as smoking cessation, alcohol prevention, medication adherence, and dietary improvements [19,20,47-50]. The educational element of this intervention portrays the capability component, which refers to the individual’s capacity to engage in necessary thought process, comprehension, and reasoning to perform the target behavior to promote disease prevention [45,51]. By imparting knowledge about the consequences of specific health behaviors and fostering an understanding of preventative measures, health education interventions aim to bridge the gap between awareness and action [52]. Previous studies have reported that education-based interventions have been the most successful in increasing vaccine uptake and have influenced different behavior in subsequent seasons as well [28,53]. By leveraging mobile health technologies and their easy accessibility, these interventions can reach larger populations, ultimately leading to a greater public health impact. Digital education is a broad construct describing a wide range of teaching and learning strategies [54] that has the potential to reduce the constraints of time and geographic barriers by allowing access to educational materials without restrictions. Considering these benefits and according to several systematic reviews of digital educational programs, it was found that these interventions had a favorable effect in terms of cost as well as health outcomes [55-57].

Nudge messages influence the timing of delivering specific education. Our findings indicated that nudge messages with educational content or a call to action can have a meaningful impact on people’s behavior. Previous research has highlighted the potential of mobile health interventions to provide effective and scalable [58] interventions for improving health outcomes including getting vaccinated [5,35]. The Behavioral Insights Team (popularly known as the “Nudge Unit”) was established by the UK government in 2010 as the first government body applying behavioral science to policy [59]. Recently, the NUDGE-FLU (Nationwide Utilization of Danish Government Electronic Letter System For Increasing Influenza Vaccine Uptake) trial found that letters designed using behavioral science principles and delivered through a governmental electronic letter system were effective in increasing influenza vaccination rates among older adults in Denmark [60]. Specifically in diabetes and metabolic chronic conditions, the association between engagement with digital health platforms and improved clinical outcomes was demonstrated [61,62]. Digital nudges represent the “opportunity” aspect in the COM-B model for behavior change [20]. They gently steer individuals toward healthier or more beneficial behaviors without being forceful and improve decision-making. Digital health interventions hold promise in addressing health disparities by providing accessible information, as evidenced by the demonstrated improvements in glycemic outcomes across different racial groups [63].

Despite the reported beneficial effects of influenza vaccination in people with diabetes [64], a substantial portion of them remain unvaccinated. It has been previously found that the most effective interventions were based largely on knowledge and awareness raising that were tailored to specific populations [65]. Digital health intervention with nudge messages can be tailored to the individual’s specific needs and circumstances, making the guidance more relevant and effective. A personalized intervention embodies the “motivation” component in the COM-B model for behavior change [20], which is known as the internal process that influences behaviors [66]. A dynamic personalized approach to developing persuasive technologies is essential to encourage the users to continue managing their health, and moreover to change their perspective or take new actions in improving their condition.

The digital platform for multicondition management represents an opportunity for people with diabetes to improve the self-management of their disease by monitoring their blood glucose, blood pressure, and weight, logging their meals and medications, and seeking advice using educational content or coaching sessions. Personalized digital health holds the potential to use gathered data and tailor interventions based on individual patient needs [67]. Among nonclinical populations, digital health platforms have increasingly been used to facilitate healthy behavior change in a variety of domains, including sleep, physical activity, diet and tobacco use [68]. Previous research on the same platform has demonstrated the capability of this digital approach to personalize the service according to different racial or ethnic groups’ unique needs [63]. Another study has investigated the personalized efficacy of this digital therapeutic for pain management, tailored to users’ characteristics [69].

This study acknowledges the importance of implementing digital tools to bridge the gap between research and practice and contributed in several key areas [26,70-73].

It assesses how well the interventions were implemented in real-world settings, providing evidence on their effectiveness and feasibility. Moreover, the study provided data on necessary adaptations to flu vaccination interventions to fit specific contexts or populations, enhancing their applicability and generated knowledge that can inform policy decisions and best practices, promoting evidence-based approaches in health care and other fields. The population considered in this study enhanced generalizability (people with type 1 and those with type 2) and examined the scalability and accessibility contributing to broader public health impact. Finally, it focused on strategies to optimize the intervention tools, ultimately aiming to improve awareness of health condition and risks through effective intervention delivery. Main reasons for not receiving the influenza vaccine were perceptions of not being at-risk, or not thinking of it [16]. Using the Reach, Effectiveness, Adoption, Implementation, and Maintenance (RE-AIM) [74,75] framework to evaluate the impact of the adapted intervention in this study, incorporating the flu nudge intervention into an existing diabetes digital therapeutics platform can be an effective strategy to promote flu vaccination uptake in an high-risk population, improving the ability to implement this important public health initiative. Reflecting on this in more detail across the 5 dimensions of RE-AIM:

- Reach for the flu nudge intervention is broader, benefiting from the growing user base of the diabetes digital therapeutics platform, that meets patients where they are on their smartphones and is not an isolated flu vaccination campaign requiring dedicated effort to reach the target audience.
- The effectiveness of the flu nudge intervention adapted to the character of the digital platform trended better than implementing the intervention the same way as it was originally studied.
- Adoption likelihood is optimized in a conducive environment with the flu nudge part of the total personalized engagement experience of the digital platform, where education on the risk of flu is provided in context to the user’s diabetes status and other health metrics monitored on the same platform that provides a whole-person care perspective.
- Implementation of the adapted flu nudge remained true to the core intervention principles and adapted the frequency and cadence of intervention in line with the digital platform’s operating mechanism for personalized interventions.
- Maintenance observation is made possible via the same digital platform over time provided the user stays engaged with the platform, which is designed to sustain engagement and has been associated with improved clinical measures in diabetes after 6 months [76], 12 months and 2 and 3 years [63,77,78].

### Strengths and Limitations

This study observed the flu vaccination intent and behavior of active users of the platform with diabetes, as part of their routine journey and not as part of a controlled protocol that dictated specific actions which could introduce bias. The T1D to T2D ratio closely matches that of T1D and T2D prevalence in the general population.

Compared with previous studies, which had an average baseline vaccination rate of around 60% [5,35], our control group demonstrated a higher rate of 70.5%. This presents a significant challenge to improve upon, as it indicates our initial cohort was already highly motivated in their health. This higher baseline may have made it more challenging to achieve further improvement, highlighting the robustness of our study’s outcomes. Moreover, no financial incentives were provided that could have biased toward vaccination uptake, in contrast to other similar studies [5,35]. The positive association between knowledge retention on the awareness of risk to vaccination rate provided insight to a mediator of the result and provided one possible explanation to the mechanism by which the adapted flu nudge intervention led to a higher flu vaccination rate. Other mediators and the relative strength of mediators can be topics for future research.

There were certain limitations to the study. Given this was performed in a real-world setting it was not possible to assess the reasons for noncompletion of surveys from survey 1 at time 0 to survey 3 at month 6 and vaccination rates could only be based on completers which differed slightly from survey 2 to survey 3. This also reflects a limitation on the cross-sectional nature of this study compared with a longitudinal study which could have generated more insights. Moreover, due to the survey response rate of approximately 13% for surveys 2 or 3, the integrity of findings by reducing sample size and potentially biasing results if not handled properly. In this study we use the complete case analysis in handling the missing data because it avoids imputation assumptions and preserves the observed data’s integrity, minimizing the risk of biases into the analysis, ensuring robustness in drawing conclusions about the intervention’s effectiveness and feasibility. The magnitude of the difference in vaccination rate was small and not statistically significant, which could have been due to a high vaccination rate at baseline. There was also a noticeable difference in the average age between completers and noncompleters, and older adults tend to be more aware of their health status, which could limit the generalizability for a younger age group.

### Conclusion

Implementation of the digital flu nudge intervention from a RCT into a personalized digital diabetes management platform improved awareness and knowledge retention on flu vaccination risk and trended toward higher vaccination rates compared with the RCT and the control group. The higher awareness of flu risk in people with diabetes was associated with higher flu vaccination rates, demonstrating the added benefit of the digital platform to facilitate people with diabete’s behavior change from awareness to action on their health. This study suggests that incorporating evidence-based interventions into digital chronic disease management platforms may be an effective strategy to increase the uptake of proven interventions in real-world settings, as well a potential strategy for the scale-up of evidence-based interventions to manage population health. This study follows the CONSORT-EHEALTH guidelines (Multimedia Appendix 3).

## Data Availability

The datasets generated during and/or analyzed during this study are not publicly available due to company privacy policy but are available from the corresponding author on reasonable request according to the subject to company policies. Requests to access the datasets should be directed to yifat@dariohealth.com.

## Appendix

Multimedia Appendix 1 Baseline survey.

Multimedia Appendix 2 Three- and/or six-month survey.

Multimedia Appendix 3 CONSORT-eHEALTH checklist (V 1.6.1).

## Abbreviations

CDC: Center for Disease Control and Prevention
COM-B: Capability, Opportunity, Motivation, and Behavior
NCQA: US National Committee for Quality Assurance
OR: odds ratio
RCT: randomized controlled trial
RE-AIM: Reach, Effectiveness, Adoption, Implementation, Maintenance
WHO: World Health Organization
