# Supplementary figures and images for: Digital Health Intervention on Awareness of Vaccination Against Influenza Among Adults With Diabetes: Pragmatic Randomized Follow-Up Study
Source: J Med Internet Res. 2025 Apr 10;27:e68936. doi: 10.2196/68936 (PMC12022521; doi:10.2196/68936)

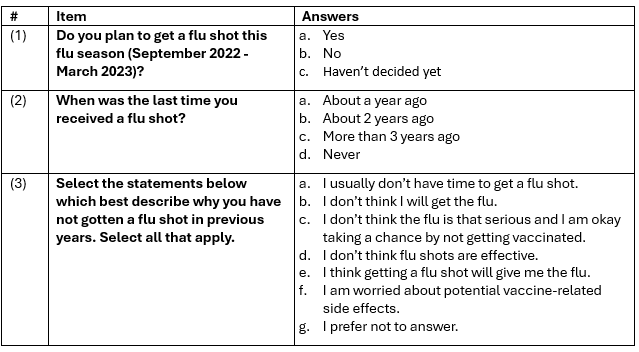

Supplement: Multimedia Appendix 1 [file jmir_v27i1e68936_app1.png]

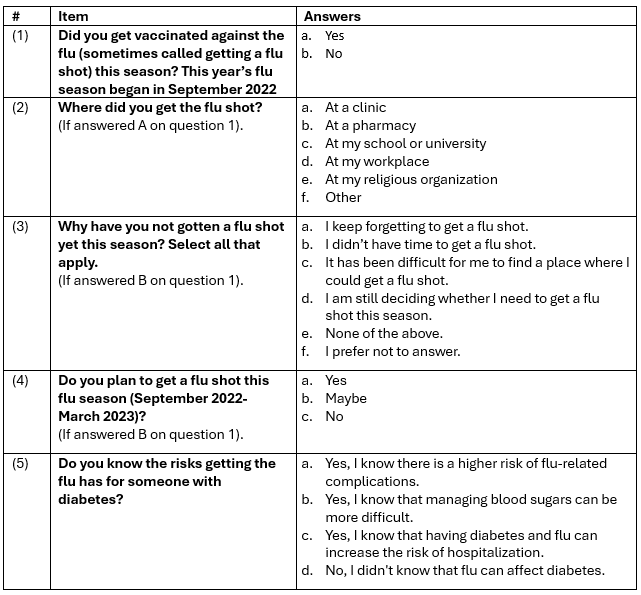

Supplement: Multimedia Appendix 2 [file jmir_v27i1e68936_app2.png]
