# Supplementary material for: Digital Health Intervention on Awareness of Vaccination Against Influenza Among Adults With Diabetes: Pragmatic Randomized Follow-Up Study
Source: J Med Internet Res. 2025 Apr 10;27:e68936. doi: 10.2196/68936 (PMC12022521; doi:10.2196/68936)
Supplement: Multimedia Appendix 3 [file jmir_v27i1e68936_app3.pdf]

CONSORT-EHEALTH (V 1.6.1) - Submission/Publication Form

The CONSORT-EHEALTH checklist is intended for authors of randomized trials evaluating web-based and Internet-based applications/interventions, including mobile interventions, electronic games (incl multiplayer games), social media, certain telehealth applications, and other interactive and/or networked electronic applications. Some of the items (e.g. all subitems under item 5 - description of the intervention) may also be applicable for other study designs.

The goal of the CONSORT EHEALTH checklist and guideline is to be
a) a guide for reporting for authors of RCTs,
b) to form a basis for appraisal of an ehealth trial (in terms of validity)

CONSORT-EHEALTH items/subitems are MANDATORY reporting items for studies published in the Journal of Medical Internet Research and other journals / scientific societies endorsing the checklist.

Items numbered 1., 2., 3., 4a., 4b etc are original CONSORT or CONSORT-NPT (non-pharmacologic treatment) items.
Items with Roman numerals (i., ii, iii, iv etc.) are CONSORT-EHEALTH extensions/clarifications.

As the CONSORT-EHEALTH checklist is still considered in a formative stage, we would ask that you also RATE ON A SCALE OF 1-5 how important/useful you feel each item is FOR THE PURPOSE OF THE CHECKLIST and reporting guideline (optional).

Mandatory reporting items are marked with a red *.
In the textboxes, either copy & paste the relevant sections from your manuscript into this form - please include any quotes from your manuscript in QUOTATION MARKS,
or answer directly by providing additional information not in the manuscript, or elaborating on why the item was not relevant for this study.

YOUR ANSWERS WILL BE PUBLISHED AS A SUPPLEMENTARY FILE TO YOUR PUBLICATION IN JMIR AND ARE CONSIDERED PART OF YOUR PUBLICATION (IF ACCEPTED).
Please fill in these questions diligently. Information will not be copyedited, so please use proper spelling and grammar, use correct capitalization, and avoid abbreviations.

DO NOT FORGET TO SAVE AS PDF _AND_ CLICK THE SUBMIT BUTTON SO YOUR ANSWERS ARE IN OUR DATABASE !!!

Citation Suggestion (if you append the pdf as Appendix we suggest to cite this paper in the caption):
Eysenbach G, CONSORT-EHEALTH Group
CONSORT-EHEALTH: Improving and Standardizing Evaluation Reports of Web-based and Mobile Health Interventions
J Med Internet Res 2011;13(4):e126
URL: http://www.jmir.org/2011/4/e126/
doi: 10.2196/jmir.1923
PMID: 22209829

yifath2004@gmail.com החלפת חשבון

לא בשיתוף

הטיוטה נשמרה

כוכבית (*) מציינת שאלה שאי אפשר לדלג עליה

* Your name

First Last

Yifat Fundoiano-Hershcovitz

* Primary Affiliation (short), City, Country

University of Toronto, Toronto, Canada

Dario Health, Caesarea, Israel

* Your e-mail address

abc@gmail.com

yifat@dariohealth.com

* Title of your manuscript

.Provide the (draft) title of your manuscript

Digital Health Intervention on Awareness of Vaccination Against Influenza Among Adults With Diabetes: Pragmatic Randomized Follow-Up Study

* Name of your App/Software/Intervention

If there is a short and a long/alternate name, write the short name first and add the long .name in brackets

Dario App

Evaluated Version (if any)

"e.g. "V1", "Release 2017-03-01", "Version 2.0.27913

התשובה שלך

* Language(s)

What language is the intervention/app in? If multiple languages are available, separate by comma (e.g. "English, French")

English

URL of your Intervention Website or App

e.g. a direct link to the mobile app on app in appstore (itunes, Google Play), or URL of the .website. If the intervention is a DVD or hardware, you can also link to an Amazon page

התשובה שלך

URL of an image/screenshot (optional)

התשובה שלך

* Accessibility

?Can an enduser access the intervention presently

- access is free and open
- access only for special usergroups, not open
- access is open to everyone, but requires payment/subscription/in-app purchases
- app/intervention no longer accessible
- (selected) אחר: The Dario app is accessible

* Primary Medical Indication/Disease/Condition

e.g. "Stress", "Diabetes", or define the target group in brackets after the condition, e.g. ""Autism (Parents of children with)", "Alzheimers (Informal Caregivers of)

Diabetes

* Primary Outcomes measured in trial

comma-separated list of primary outcomes reported in the trial

the difference in vaccination rates between the

Secondary/other outcomes

?Are there any other outcomes the intervention is expected to affect

Increased health risk awareness

* "Recommended "Dose

?What do the instructions for users say on how often the app should be used

- ○ Approximately Daily
- ○ Approximately Weekly
- ○ Approximately Monthly
- ○ Approximately Yearly
- ○ "as needed"
- ● אחר: The 3 groups defined in the study: group A received flu nudge message

* Approx. Percentage of Users (starters) still using the app as recommended after 3 months

- unknown / not evaluated
- 0-10%
- 11-20%
- 21-30%
- 31-40%
- 41-50%
- 51-60%
- 61-70%
- 71%-80%
- 81-90%
- 91-100%
- (selected) אחר: The percentage still using the Flu nudge intervention is not evaluated cı

* ?Overall, was the app/intervention effective

- ( ) yes: all primary outcomes were significantly better in intervention group vs control
- ( ) partly: SOME primary outcomes were significantly better in intervention group vs control
- ( ) no statistically significant difference between control and intervention
- ( ) potentially harmful: control was significantly better than intervention in one or more outcomes
- ( ) inconclusive: more research is needed
- (x) אחר: Vaccination rates were 71.0% in group A, 71.9% in group B, and 70.5% ir

* Article Preparation Status/Stage

At which stage in your article preparation are you currently (at the time you fill in this form)

- ○ not submitted yet - in early draft status
- ○ not submitted yet - in late draft status, just before submission
- ○ submitted to a journal but not reviewed yet
- ○ submitted to a journal and after receiving initial reviewer comments
- ◉ submitted to a journal and accepted, but not published yet
- ○ published
- ○ אחר:

* Journal

If you already know where you will submit this paper (or if it is already submitted), please provide the journal name (if it is not JMIR, provide the journal name under "other")

- not submitted yet / unclear where I will submit this
- Journal of Medical Internet Research (JMIR) (selected)
- JMIR mHealth and UHealth
- JMIR Serious Games
- JMIR Mental Health
- JMIR Public Health
- JMIR Formative Research
- Other JMIR sister journal
- אחר:

* ?Is this a full powered effectiveness trial or a pilot/feasibility trial

- Pilot/feasibility (selected)
- Fully powered

* Manuscript tracking number

If this is a JMIR submission, please provide the manuscript tracking number under "other" (The ms tracking number can be found in the submission acknowledgement email, or when you login as author in JMIR. If the paper is already published in JMIR, then the ms tracking number is the four-digit number at the end of the DOI, to be found at the bottom of each published article in JMIR)

- ○ no ms number (yet) / not (yet) submitted to / published in JMIR
- ◉ אחר: JMIR ms#68936

TITLE AND ABSTRACT

1a) TITLE: Identification as a randomized trial in the title

* ?1a) Does your paper address CONSORT item 1a

I.e does the title contain the phrase "Randomized Controlled Trial"? (if not, explain the reason under "other")

- ○ yes
- ◉ אחר: Pragmatic Randomized Follow-Up Study as it evaluates how well the int

1a-i) Identify the mode of delivery in the title

Identify the mode of delivery. Preferably use “web-based” and/or “mobile” and/or “electronic game” in the title. Avoid ambiguous terms like “online”, “virtual”, “interactive”. Use “Internet-based” only if Intervention includes non-web-based Internet components (e.g. email), use “computer-based” or “electronic” only if offline products are used. Use “virtual” only in the context of “virtual reality” (3-D worlds). Use “online” only in the context of “online support groups”. Complement or substitute product names with broader terms for the class of products (such as “mobile” or “smart phone” instead of “iphone”), especially if the .application runs on different platforms

	5	4	3	2	1	
essential	○	○	○	○	○	subitem not at all important

* ?Does your paper address subitem 1a-i

Copy and paste relevant sections from manuscript title (include quotes in quotation marks "like this" to indicate direct quotes from your manuscript), or elaborate on this item by providing additional information not in the ms, or briefly explain why the item is not applicable/relevant for your study

The 1a-i item is relevant for the study and was addressed during the copyediting process.

1a-ii) Non-web-based components or important co-interventions in title

Mention non-web-based components or important co-interventions in title, if any (e.g., .“with telephone support”)

	5	4	3	2	1	
essential	○	○	○	○	○	subitem not at all important

?Does your paper address subitem 1a-ii

Copy and paste relevant sections from manuscript title (include quotes in quotation marks "like this" to indicate direct quotes from your manuscript), or elaborate on this item by providing additional information not in the ms, or briefly explain why the item is not applicable/relevant for your study

התשובה שלך

1a-iii) Primary condition or target group in the title

Mention primary condition or target group in the title, if any (e.g., “for children with Type I Diabetes”) Example: A Web-based and Mobile Intervention with Telephone Support for Children with Type I Diabetes: Randomized Controlled Trial

	5	4	3	2	1	
essential	○	○	○	○	○	subitem not at all important

* ?Does your paper address subitem 1a-iii

Copy and paste relevant sections from manuscript title (include quotes in quotation marks "like this" to indicate direct quotes from your manuscript), or elaborate on this item by providing additional information not in the ms, or briefly explain why the item is not applicable/relevant for your study

Item 1a-iii is relevant to the study, as it pertains to the target population: individuals with diabetes that can receive the flu vaccination

1b) ABSTRACT: Structured summary of trial design, methods, results, and conclusions

NPT extension: Description of experimental treatment, comparator, care providers, centers, .and blinding status

1b-i) Key features/functionalities/components of the intervention and comparator in the METHODS section of the ABSTRACT

Mention key features/functionalities/components of the intervention and comparator in the abstract. If possible, also mention theories and principles used for designing the site. Keep in mind the needs of systematic reviewers and indexers by including important synonyms. (Note: Only report in the abstract what the main paper is reporting. If this information is missing from the main body of text, consider adding it)

	5	4	3	2	1	
essential	○	○	○	○	○	subitem not at all important

* ?Does your paper address subitem 1b-i

Copy and paste relevant sections from the manuscript abstract (include quotes in quotation marks "like this" to indicate direct quotes from your manuscript), or elaborate on this item by providing additional information not in the ms, or briefly explain why the item is not applicable/relevant for your study

The item is relevant for the study key components of the intervention and comparator are described in the abstract

1b-ii) Level of human involvement in the METHODS section of the ABSTRACT

Clarify the level of human involvement in the abstract, e.g., use phrases like “fully automated” vs. “therapist/nurse/care provider/physician-assisted” (mention number and expertise of providers involved, if any). (Note: Only report in the abstract what the main paper is reporting. If this information is missing from the main body of text, consider adding it)

	5	4	3	2	1	
essential	○	○	○	○	○	subitem not at all important

?Does your paper address subitem 1b-ii

Copy and paste relevant sections from the manuscript abstract (include quotes in quotation marks "like this" to indicate direct quotes from your manuscript), or elaborate on this item by providing additional information not in the ms, or briefly explain why the item is not applicable/relevant for your study

התשובה שלך

1b-iii) Open vs. closed, web-based (self-assessment) vs. face-to-face assessments in the METHODS section of the ABSTRACT

Mention how participants were recruited (online vs. offline), e.g., from an open access website or from a clinic or a closed online user group (closed usergroup trial), and clarify if this was a purely web-based trial, or there were face-to-face components (as part of the intervention or for assessment). Clearly say if outcomes were self-assessed through questionnaires (as common in web-based trials). Note: In traditional offline trials, an open trial (open-label trial) is a type of clinical trial in which both the researchers and participants know which treatment is being administered. To avoid confusion, use “blinded” or “unblinded” to indicated the level of blinding instead of “open”, as “open” in web-based trials usually refers to “open access” (i.e. participants can self-enrol). (Note: Only report in the abstract what the main paper is reporting. If this information is missing from the main body of text, consider adding it)

	5	4	3	2	1	
essential	○	○	○	○	○	subitem not at all important

?Does your paper address subitem 1b-iii

Copy and paste relevant sections from the manuscript abstract (include quotes in quotation marks "like this" to indicate direct quotes from your manuscript), or elaborate on this item by providing additional information not in the ms, or briefly explain why the item is not applicable/relevant for your study

התשובה שלך

1b-iv) RESULTS section in abstract must contain use data

Report number of participants enrolled/assessed in each group, the use/uptake of the intervention (e.g., attrition/adherence metrics, use over time, number of logins etc.), in addition to primary/secondary outcomes. (Note: Only report in the abstract what the main paper is reporting. If this information is missing from the main body of text, consider adding it)

	5	4	3	2	1	
essential	○	○	○	○	○	subitem not at all important

?Does your paper address subitem 1b-iv

Copy and paste relevant sections from the manuscript abstract (include quotes in quotation marks "like this" to indicate direct quotes from your manuscript), or elaborate on this item by providing additional information not in the ms, or briefly explain why the item is not applicable/relevant for your study

התשובה שלך

1b-v) CONCLUSIONS/DISCUSSION in abstract for negative trials

Conclusions/Discussions in abstract for negative trials: Discuss the primary outcome - if the trial is negative (primary outcome not changed), and the intervention was not used, discuss whether negative results are attributable to lack of uptake and discuss reasons. (Note: Only report in the abstract what the main paper is reporting. If this information is missing from the main body of text, consider adding it)

	5	4	3	2	1	
essential	○	○	○	○	○	subitem not at all important

?Does your paper address subitem 1b-v

Copy and paste relevant sections from the manuscript abstract (include quotes in quotation marks "like this" to indicate direct quotes from your manuscript), or elaborate on this item by providing additional information not in the ms, or briefly explain why the item is not applicable/relevant for your study

התשובה שלך

INTRODUCTION

2a) In INTRODUCTION: Scientific background and explanation of rationale

2a-i) Problem and the type of system/solution

Describe the problem and the type of system/solution that is object of the study: intended as stand-alone intervention vs. incorporated in broader health care program? Intended for a particular patient population? Goals of the intervention, e.g., being more cost-effective to other interventions, replace or complement other solutions? (Note: Details about the intervention are provided in “Methods” under 5)

	5	4	3	2	1	
essential	○	○	○	○	○	subitem not at all important

* ?Does your paper address subitem 2a-i

Copy and paste relevant sections from the manuscript (include quotes in quotation marks "like this" to indicate direct quotes from your manuscript), or elaborate on this item by providing additional information not in the ms, or briefly explain why the item is not applicable/relevant for your study

Item 2 ai is addressed with describing the problem and the solution in the introduction section

2a-ii) Scientific background, rationale: What is known about the (type of) system

Scientific background, rationale: What is known about the (type of) system that is the object of the study (be sure to discuss the use of similar systems for other conditions/diagnoses, if appropiate), motivation for the study, i.e. what are the reasons for and what is the context for this specific study, from which stakeholder viewpoint is the study performed, potential impact of findings [2]. Briefly justify the choice of the .comparator

	5	4	3	2	1	
essential	(●)	○	○	○	○	subitem not at all important

ניקוי הבחירה

* ?Does your paper address subitem 2a-ii

Copy and paste relevant sections from the manuscript (include quotes in quotation marks "like this" to indicate direct quotes from your manuscript), or elaborate on this item by providing additional information not in the ms, or briefly explain why the item is not applicable/relevant for your study

The item is applicable, the motivation for the study and the reason for and the context for this specific study, are well described.

2b) In INTRODUCTION: Specific objectives or hypotheses

* ?Does your paper address CONSORT subitem 2b

Copy and paste relevant sections from the manuscript (include quotes in quotation marks "like this" to indicate direct quotes from your manuscript), or elaborate on this item by providing additional information not in the ms, or briefly explain why the item is not applicable/relevant for your study

Yes the manuscript contains subitem 2b, specific objectives and hypothesis in described in the Introduction

METHODS

3a) Description of trial design (such as parallel, factorial) including allocation ratio

* ?Does your paper address CONSORT subitem 3a

Copy and paste relevant sections from the manuscript (include quotes in quotation marks "like this" to indicate direct quotes from your manuscript), or elaborate on this item by providing additional information not in the ms, or briefly explain why the item is not applicable/relevant for your study

The study design is described in the Methods section

3b) Important changes to methods after trial commencement (such as eligibility criteria), with reasons

* ?Does your paper address CONSORT subitem 3b

Copy and paste relevant sections from the manuscript (include quotes in quotation marks "like this" to indicate direct quotes from your manuscript), or elaborate on this item by providing additional information not in the ms, or briefly explain why the item is not applicable/relevant for your study

Item 3b is not relevant as the analysis performed retrospectively after the study was ended and the data was collected. A quotation from the manuscript: "The study leveraged the Dario (Dario Health Crop) digital health platform to retrospectively analyze data from 64,904 users with diabetes assigned by the platform into three groups"

3b-i) Bug fixes, Downtimes, Content Changes

Bug fixes, Downtimes, Content Changes: ehealth systems are often dynamic systems. A description of changes to methods therefore also includes important changes made on the intervention or comparator during the trial (e.g., major bug fixes or changes in the functionality or content) (5-iii) and other “unexpected events” that may have influenced .study design such as staff changes, system failures/downtimes, etc. [2]

	5	4	3	2	1	
essential	○	○	○	○	○	subitem not at all important

?Does your paper address subitem 3b-i

Copy and paste relevant sections from the manuscript (include quotes in quotation marks "like this" to indicate direct quotes from your manuscript), or elaborate on this item by providing additional information not in the ms, or briefly explain why the item is not applicable/relevant for your study

התשובה שלך

4a) Eligibility criteria for participants

* ?Does your paper address CONSORT subitem 4a

Copy and paste relevant sections from the manuscript (include quotes in quotation marks "like this" to indicate direct quotes from your manuscript), or elaborate on this item by providing additional information not in the ms, or briefly explain why the item is not applicable/relevant for your study

The item 4a is applicable and the eligibility criteria are described in the text.

4a-i) Computer / Internet literacy

Computer / Internet literacy is often an implicit “de facto” eligibility criterion - this should be .explicitly clarified

	5	4	3	2	1	
essential	○	○	○	○	○	subitem not at all important

?Does your paper address subitem 4a-i

Copy and paste relevant sections from the manuscript (include quotes in quotation marks "like this" to indicate direct quotes from your manuscript), or elaborate on this item by providing additional information not in the ms, or briefly explain why the item is not applicable/relevant for your study

התשובה שלך

:4a-ii) Open vs. closed, web-based vs. face-to-face assessments

Open vs. closed, web-based vs. face-to-face assessments: Mention how participants were recruited (online vs. offline), e.g., from an open access website or from a clinic, and clarify if this was a purely web-based trial, or there were face-to-face components (as part of the intervention or for assessment), i.e., to what degree got the study team to know the participant. In online-only trials, clarify if participants were quasi-anonymous and whether having multiple identities was possible or whether technical or logistical measures (e.g., .cookies, email confirmation, phone calls) were used to detect/prevent these

	5	4	3	2	1	
essential	○	○	○	○	○	subitem not at all important

* ?Does your paper address subitem 4a-ii

Copy and paste relevant sections from the manuscript (include quotes in quotation marks "like this" to indicate direct quotes from your manuscript), or elaborate on this item by providing additional information not in the ms, or briefly explain why the item is not applicable/relevant for your study

Item 4a-ii is applicable, the recruitment was described in the text.

4a-iii) Information giving during recruitment

Information given during recruitment. Specify how participants were briefed for recruitment and in the informed consent procedures (e.g., publish the informed consent documentation as appendix, see also item X26), as this information may have an effect on .user self-selection, user expectation and may also bias results

	5	4	3	2	1	
essential	○	○	○	○	○	subitem not at all important

?Does your paper address subitem 4a-iii

Copy and paste relevant sections from the manuscript (include quotes in quotation marks "like this" to indicate direct quotes from your manuscript), or elaborate on this item by providing additional information not in the ms, or briefly explain why the item is not applicable/relevant for your study

התשובה שלך

4b) Settings and locations where the data were collected

* ?Does your paper address CONSORT subitem 4b

Copy and paste relevant sections from the manuscript (include quotes in quotation marks "like this" to indicate direct quotes from your manuscript), or elaborate on this item by providing additional information not in the ms, or briefly explain why the item is not applicable/relevant for your study

Item 4b is applicable, the description of data collected is provided in the text.

4b-i) Report if outcomes were (self-)assessed through online questionnaires

Clearly report if outcomes were (self-)assessed through online questionnaires (as .common in web-based trials) or otherwise

	5	4	3	2	1	
essential	○	○	○	○	○	subitem not at all important

* ?Does your paper address subitem 4b-i

Copy and paste relevant sections from the manuscript (include quotes in quotation marks "like this" to indicate direct quotes from your manuscript), or elaborate on this item by providing additional information not in the ms, or briefly explain why the item is not applicable/relevant for your study

Item 4b-i is applicable, it is reported in the text how the outcomes were (self-)assessed through online surveys.

4b-ii) Report how institutional affiliations are displayed

Report how institutional affiliations are displayed to potential participants [on ehealth media], as affiliations with prestigious hospitals or universities may affect volunteer rates, use, and reactions with regards to an intervention.(Not a required item – describe only if this may bias results)

	5	4	3	2	1	
essential	○	○	○	○	○	subitem not at all important

?Does your paper address subitem 4b-ii

Copy and paste relevant sections from the manuscript (include quotes in quotation marks "like this" to indicate direct quotes from your manuscript), or elaborate on this item by providing additional information not in the ms, or briefly explain why the item is not applicable/relevant for your study

התשובה שלך

The interventions for each group with sufficient details to allow replication, (5 including how and when they were actually administered

i) Mention names, credential, affiliations of the developers, sponsors, and owners-5

Mention names, credential, affiliations of the developers, sponsors, and owners [6] (if authors/evaluators are owners or developer of the software, this needs to be declared in a .“Conflict of interest” section or mentioned elsewhere in the manuscript)

	5	4	3	2	1	
essential	○	○	○	○	○	subitem not at all important

?Does your paper address subitem 5-i

Copy and paste relevant sections from the manuscript (include quotes in quotation marks "like this" to indicate direct quotes from your manuscript), or elaborate on this item by providing additional information not in the ms, or briefly explain why the item is not applicable/relevant for your study

התשובה שלך

ii) Describe the history/development process-5

Describe the history/development process of the application and previous formative evaluations (e.g., focus groups, usability testing), as these will have an impact on .adoption/use rates and help with interpreting results

	5	4	3	2	1	
essential	○	○	○	○	○	subitem not at all important

?Does your paper address subitem 5-ii

Copy and paste relevant sections from the manuscript (include quotes in quotation marks "like this" to indicate direct quotes from your manuscript), or elaborate on this item by providing additional information not in the ms, or briefly explain why the item is not applicable/relevant for your study

התשובה שלך

iii) Revisions and updating-5

Revisions and updating. Clearly mention the date and/or version number of the application/intervention (and comparator, if applicable) evaluated, or describe whether the intervention underwent major changes during the evaluation process, or whether the development and/or content was “frozen” during the trial. Describe dynamic components such as news feeds or changing content which may have an impact on the replicability of .the intervention (for unexpected events see item 3b)

	5	4	3	2	1	
essential	○	○	○	○	○	subitem not at all important

?Does your paper address subitem 5-iii

Copy and paste relevant sections from the manuscript (include quotes in quotation marks "like this" to indicate direct quotes from your manuscript), or elaborate on this item by providing additional information not in the ms, or briefly explain why the item is not applicable/relevant for your study

התשובה שלך

iv) Quality assurance methods-5

Provide information on quality assurance methods to ensure accuracy and quality of .information provided [1], if applicable

	5	4	3	2	1	
essential	○	○	○	○	○	subitem not at all important

?Does your paper address subitem 5-iv

Copy and paste relevant sections from the manuscript (include quotes in quotation marks "like this" to indicate direct quotes from your manuscript), or elaborate on this item by providing additional information not in the ms, or briefly explain why the item is not applicable/relevant for your study

התשובה שלך

v) Ensure replicability by publishing the source code, and/or providing-5 screenshots/screen-capture video, and/or providing flowcharts of the algorithms used

Ensure replicability by publishing the source code, and/or providing screenshots/screen-capture video, and/or providing flowcharts of the algorithms used. Replicability (i.e., other researchers should in principle be able to replicate the study) is a hallmark of scientific .reporting

	5	4	3	2	1	
essential	○	○	○	○	○	subitem not at all important

?Does your paper address subitem 5-v

Copy and paste relevant sections from the manuscript (include quotes in quotation marks "like this" to indicate direct quotes from your manuscript), or elaborate on this item by providing additional information not in the ms, or briefly explain why the item is not applicable/relevant for your study

התשובה שלך

vi) Digital preservation-5

Digital preservation: Provide the URL of the application, but as the intervention is likely to change or disappear over the course of the years; also make sure the intervention is archived (Internet Archive, webcitation.org, and/or publishing the source code or screenshots/videos alongside the article). As pages behind login screens cannot be archived, consider creating demo pages which are accessible without login.

	5	4	3	2	1	
essential	○	○	○	○	○	subitem not at all important

?Does your paper address subitem 5-vi

Copy and paste relevant sections from the manuscript (include quotes in quotation marks "like this" to indicate direct quotes from your manuscript), or elaborate on this item by providing additional information not in the ms, or briefly explain why the item is not applicable/relevant for your study

התשובה שלך

vii) Access-5

Access: Describe how participants accessed the application, in what setting/context, if they had to pay (or were paid) or not, whether they had to be a member of specific group. If known, describe how participants obtained “access to the platform and Internet” [1]. To ensure access for editors/reviewers/readers, consider to provide a “backdoor” login account or demo mode for reviewers/readers to explore the application (also important for .archiving purposes, see vi)

	5	4	3	2	1	
essential	○	○	○	○	○	subitem not at all important

* ?Does your paper address subitem 5-vii

Copy and paste relevant sections from the manuscript (include quotes in quotation marks "like this" to indicate direct quotes from your manuscript), or elaborate on this item by providing additional information not in the ms, or briefly explain why the item is not applicable/relevant for your study

Item 5-vii is applicable, it is described how the users accessed the application.

viii) Mode of delivery, features/functionalities/components of the intervention and-5 comparator, and the theoretical framework

Describe mode of delivery, features/functionalities/components of the intervention and comparator, and the theoretical framework [6] used to design them (instructional strategy [1], behaviour change techniques, persuasive features, etc., see e.g., [7, 8] for terminology). This includes an in-depth description of the content (including where it is coming from and who developed it) [1],” whether [and how] it is tailored to individual circumstances and allows users to track their progress and receive feedback” [6]. This also includes a description of communication delivery channels and – if computer-mediated communication is a component – whether communication was synchronous or asynchronous [6]. It also includes information on presentation strategies [1], including page design principles, average amount of text on pages, presence of hyperlinks to other .resources, etc. [1]

	5	4	3	2	1	
essential	○	○	○	○	○	subitem not at all important

* ?Does your paper address subitem 5-viii

Copy and paste relevant sections from the manuscript (include quotes in quotation marks "like this" to indicate direct quotes from your manuscript), or elaborate on this item by providing additional information not in the ms, or briefly explain why the item is not applicable/relevant for your study

Item 5-viii is applicable, mode of delivery is described as well as the theoretical framework and the behavioral change techniques in the digital health intervention.

ix) Describe use parameters-5

Describe use parameters (e.g., intended “doses” and optimal timing for use). Clarify what instructions or recommendations were given to the user, e.g., regarding timing, frequency, .heaviness of use, if any, or was the intervention used ad libitum

	5	4	3	2	1	
essential	○	○	○	○	○	subitem not at all important

?Does your paper address subitem 5-ix

Copy and paste relevant sections from the manuscript (include quotes in quotation marks "like this" to indicate direct quotes from your manuscript), or elaborate on this item by providing additional information not in the ms, or briefly explain why the item is not applicable/relevant for your study

התשובה שלך

x) Clarify the level of human involvement-5

Clarify the level of human involvement (care providers or health professionals, also technical assistance) in the e-intervention or as co-intervention (detail number and expertise of professionals involved, if any, as well as “type of assistance offered, the timing and frequency of the support, how it is initiated, and the medium by which the assistance is delivered”. It may be necessary to distinguish between the level of human involvement required for the trial, and the level of human involvement required for a routine application .outside of a RCT setting (discuss under item 21 – generalizability)

	5	4	3	2	1	
essential	○	○	○	○	○	subitem not at all important

?Does your paper address subitem 5-x

Copy and paste relevant sections from the manuscript (include quotes in quotation marks "like this" to indicate direct quotes from your manuscript), or elaborate on this item by providing additional information not in the ms, or briefly explain why the item is not applicable/relevant for your study

התשובה שלך

xi) Report any prompts/reminders used-5

Report any prompts/reminders used: Clarify if there were prompts (letters, emails, phone calls, SMS) to use the application, what triggered them, frequency etc. It may be necessary to distinguish between the level of prompts/reminders required for the trial, and the level of prompts/reminders for a routine application outside of a RCT setting (discuss under item .21 – generalizability)

	5	4	3	2	1	
essential	○	○	○	○	○	subitem not at all important

* ?Does your paper address subitem 5-xi

Copy and paste relevant sections from the manuscript (include quotes in quotation marks "like this" to indicate direct quotes from your manuscript), or elaborate on this item by providing additional information not in the ms, or briefly explain why the item is not applicable/relevant for your study

The item 5-xi is applicable, prompts and reminders were described and examples were provided.

xii) Describe any co-interventions (incl. training/support)-5

Describe any co-interventions (incl. training/support): Clearly state any interventions that are provided in addition to the targeted eHealth intervention, as ehealth intervention may not be designed as stand-alone intervention. This includes training sessions and support [1]. It may be necessary to distinguish between the level of training required for the trial, and the level of training for a routine application outside of a RCT setting (discuss under .item 21 – generalizability

	5	4	3	2	1	
essential	○	○	○	○	○	subitem not at all important

* ?Does your paper address subitem 5-xii

Copy and paste relevant sections from the manuscript (include quotes in quotation marks "like this" to indicate direct quotes from your manuscript), or elaborate on this item by providing additional information not in the ms, or briefly explain why the item is not applicable/relevant for your study

No-additional co-interventions were described in the text. The participants included are Dario members that were using the app and device. the device connectivity is described in the text: "This study used the Dario multicondition digital therapeutics platform for chronic conditions management including diabetes, hypertension, and obesity. The platform combines a glucometer with a smartphone app that is available for both Android and iOS devices. The blood glucose monitoring system consists of a small pocket-sized holder for strips, a lancet, and the meter. The meter is removed from the holder and plugged directly into the smartphone, effectively converting the smartphone into the display screen for the meter.

6a) Completely defined pre-specified primary and secondary outcome measures, including how and when they were assessed

* ?Does your paper address CONSORT subitem 6a

Copy and paste relevant sections from the manuscript (include quotes in quotation marks "like this" to indicate direct quotes from your manuscript), or elaborate on this item by providing additional information not in the ms, or briefly explain why the item is not applicable/relevant for your study

Item 6a is applicable, the outcomes assessments were defined

6a-i) Online questionnaires: describe if they were validated for online use and apply CHERRIES items to describe how the questionnaires were designed/deployed

If outcomes were obtained through online questionnaires, describe if they were validated for online use and apply CHERRIES items to describe how the questionnaires were .designed/deployed [9]

	5	4	3	2	1	
essential	○	○	○	○	○	subitem not at all important

?Does your paper address subitem 6a-i

Copy and paste relevant sections from manuscript text

התשובה שלך

6a-ii) Describe whether and how “use” (including intensity of use/dosage) was defined/measured/monitored

Describe whether and how “use” (including intensity of use/dosage) was defined/measured/monitored (logins, logfile analysis, etc.). Use/adoption metrics are .important process outcomes that should be reported in any ehealth trial

	5	4	3	2	1	
essential	○	○	○	○	○	subitem not at all important

?Does your paper address subitem 6a-ii

Copy and paste relevant sections from manuscript text

התשובה שלך

6a-iii) Describe whether, how, and when qualitative feedback from participants was obtained

Describe whether, how, and when qualitative feedback from participants was obtained .(e.g., through emails, feedback forms, interviews, focus groups)

	5	4	3	2	1	
essential	○	○	○	○	○	subitem not at all important

?Does your paper address subitem 6a-iii

Copy and paste relevant sections from manuscript text

התשובה שלך

6b) Any changes to trial outcomes after the trial commenced, with reasons

* ?Does your paper address CONSORT subitem 6b

Copy and paste relevant sections from the manuscript (include quotes in quotation marks "like this" to indicate direct quotes from your manuscript), or elaborate on this item by providing additional information not in the ms, or briefly explain why the item is not applicable/relevant for your study

Item 6b is not applicable as the study was performed on data that was already collected, as mentioned in the abstract: "The study leveraged the Dario (Dario Health Crop) digital health platform to retrospectively analyze data from 64,904 users with diabetes assigned by the platform into three groups:"

7a) How sample size was determined

NPT: When applicable, details of whether and how the clustering by care provides or centers was addressed

7a-i) Describe whether and how expected attrition was taken into account when calculating the sample size

Describe whether and how expected attrition was taken into account when calculating the .sample size

	5	4	3	2	1	
essential	○	○	○	○	○	subitem not at all important

?Does your paper address subitem 7a-i

Copy and paste relevant sections from manuscript title (include quotes in quotation marks "like this" to indicate direct quotes from your manuscript), or elaborate on this item by providing additional information not in the ms, or briefly explain why the item is not applicable/relevant for your study

התשובה שלך

7b) When applicable, explanation of any interim analyses and stopping guidelines

?Does your paper address CONSORT subitem 7b *

Copy and paste relevant sections from the manuscript (include quotes in quotation marks "like this" to indicate direct quotes from your manuscript), or elaborate on this item by providing additional information not in the ms, or briefly explain why the item is not applicable/relevant for your study

Item 7b is not applicable, the study ended at the time point as planned. as mentioned in the abstract: "Surveys were conducted at baseline, 3 months, and 6 months to assess vaccination status, awareness of influenza risks, and recollection of educational content." and in the Method section:"users were sent an online baseline survey before any intervention content, a midstudy assessment at 3 months, and a final assessment at 6 months"

8a) Method used to generate the random allocation sequence

NPT: When applicable, how care providers were allocated to each trial group

* ?Does your paper address CONSORT subitem 8a

Copy and paste relevant sections from the manuscript (include quotes in quotation marks "like this" to indicate direct quotes from your manuscript), or elaborate on this item by providing additional information not in the ms, or briefly explain why the item is not applicable/relevant for your study

Subitem 8a is not applicable as care providers w=did not participated in the study design

8b) Type of randomisation; details of any restriction (such as blocking and block size)

* ?Does your paper address CONSORT subitem 8b

Copy and paste relevant sections from the manuscript (include quotes in quotation marks "like this" to indicate direct quotes from your manuscript), or elaborate on this item by providing additional information not in the ms, or briefly explain why the item is not applicable/relevant for your study

Item 8b is applicable, randomization was described in the text.

Mechanism used to implement the random allocation sequence (such as (9 sequentially numbered containers), describing any steps taken to conceal the sequence until interventions were assigned

* ?Does your paper address CONSORT subitem 9

Copy and paste relevant sections from the manuscript (include quotes in quotation marks "like this" to indicate direct quotes from your manuscript), or elaborate on this item by providing additional information not in the ms, or briefly explain why the item is not applicable/relevant for your study

Item 9 is not applicable as users in that trial were automatically assigned to 3 groups via the customer engagement platform using a randomization algorithm to assign each selected user to one of the test variations.

Who generated the random allocation sequence, who enrolled participants, and (10 who assigned participants to interventions

* ?Does your paper address CONSORT subitem 10

Copy and paste relevant sections from the manuscript (include quotes in quotation marks "like this" to indicate direct quotes from your manuscript), or elaborate on this item by providing additional information not in the ms, or briefly explain why the item is not applicable/relevant for your study

Item 10 is applicable as the randomization is described in the text.

11a) If done, who was blinded after assignment to interventions (for example, participants, care providers, those assessing outcomes) and how
NPT: Whether or not administering co-interventions were blinded to group assignment

11a-i) Specify who was blinded, and who wasn’t

Specify who was blinded, and who wasn’t. Usually, in web-based trials it is not possible to blind the participants [1, 3] (this should be clearly acknowledged), but it may be possible to blind outcome assessors, those doing data analysis or those administering co-interventions (if any).

	5	4	3	2	1	
essential	○	○	○	○	○	subitem not at all important

Does your paper address subitem 11a-i? *

Copy and paste relevant sections from the manuscript (include quotes in quotation marks "like this" to indicate direct quotes from your manuscript), or elaborate on this item by providing additional information not in the ms, or briefly explain why the item is not applicable/relevant for your study

Item 11 a-i is not applicable as users in that trial were automatically assigned to 3 groups via the customer engagement platform using a randomization algorithm to assign each selected user to one of the test variations.

11a-ii) Discuss e.g., whether participants knew which intervention was the ”“intervention of interest” and which one was the “comparator

Informed consent procedures (4a-ii) can create biases and certain expectations - discuss e.g., whether participants knew which intervention was the “intervention of interest” and .”which one was the “comparator

	5	4	3	2	1	
essential	○	○	○	○	○	subitem not at all important

?Does your paper address subitem 11a-ii

Copy and paste relevant sections from the manuscript (include quotes in quotation marks "like this" to indicate direct quotes from your manuscript), or elaborate on this item by providing additional information not in the ms, or briefly explain why the item is not applicable/relevant for your study

התשובה שלך

11b) If relevant, description of the similarity of interventions

this item is usually not relevant for ehealth trials as it refers to similarity of a placebo or) (sham intervention to a active medication/intervention

* ?Does your paper address CONSORT subitem 11b

Copy and paste relevant sections from the manuscript (include quotes in quotation marks "like this" to indicate direct quotes from your manuscript), or elaborate on this item by providing additional information not in the ms, or briefly explain why the item is not applicable/relevant for your study

Item 11b is not applicable as placebo is not applicable in this study. there is a control group that did not receive any intervention.

12a) Statistical methods used to compare groups for primary and secondary outcomes

NPT: When applicable, details of whether and how the clustering by care providers or centers was addressed

* ?Does your paper address CONSORT subitem 12a

Copy and paste relevant sections from the manuscript (include quotes in quotation marks "like this" to indicate direct quotes from your manuscript), or elaborate on this item by providing additional information not in the ms, or briefly explain why the item is not applicable/relevant for your study

Item 12a is applicable and the statistical analysis used for comparison of the groups was provided in the text.

12a-i) Imputation techniques to deal with attrition / missing values

Imputation techniques to deal with attrition / missing values: Not all participants will use the intervention/comparator as intended and attrition is typically high in ehealth trials. Specify how participants who did not use the application or dropped out from the trial were treated in the statistical analysis (a complete case analysis is strongly discouraged, and .simple imputation techniques such as LOCF may also be problematic [4])

	5	4	3	2	1	
essential	○	○	○	○	○	subitem not at all important

* ?Does your paper address subitem 12a-i

Copy and paste relevant sections from the manuscript (include quotes in quotation marks "like this" to indicate direct quotes from your manuscript), or elaborate on this item by providing additional information not in the ms, or briefly explain why the item is not applicable/relevant for your study

The paper address subitem 12 a-i In this study we used the complete case analysis in handling the missing data because it avoids imputation assumptions and preserves the observed data’s integrity

12b) Methods for additional analyses, such as subgroup analyses and adjusted analyses

* ?Does your paper address CONSORT subitem 12b

Copy and paste relevant sections from the manuscript (include quotes in quotation marks "like this" to indicate direct quotes from your manuscript), or elaborate on this item by providing additional information not in the ms, or briefly explain why the item is not applicable/relevant for your study

Item 12b does not applicable. Subgroup analysis was not performed in the study.

X26) REB/IRB Approval and Ethical Considerations [recommended as subheading under "Methods"] (not a CONSORT item)

X26-i) Comment on ethics committee approval

	5	4	3	2	1	
essential	○	○	○	○	○	subitem not at all important

?Does your paper address subitem X26-i

Copy and paste relevant sections from the manuscript (include quotes in quotation marks "like this" to indicate direct quotes from your manuscript), or elaborate on this item by providing additional information not in the ms, or briefly explain why the item is not applicable/relevant for your study

התשובה שלך

x26-ii) Outline informed consent procedures

Outline informed consent procedures e.g., if consent was obtained offline or online (how? Checkbox, etc.?), and what information was provided (see 4a-ii). See [6] for some items to be included in informed consent documents.

	5	4	3	2	1	
essential	○	○	○	○	○	subitem not at all important

?Does your paper address subitem X26-ii

Copy and paste relevant sections from the manuscript (include quotes in quotation marks "like this" to indicate direct quotes from your manuscript), or elaborate on this item by providing additional information not in the ms, or briefly explain why the item is not applicable/relevant for your study

התשובה שלך

X26-iii) Safety and security procedures

Safety and security procedures, incl. privacy considerations, and any steps taken to reduce the likelihood or detection of harm (e.g., education and training, availability of a hotline)

	5	4	3	2	1	
essential	○	○	○	○	○	subitem not at all important

?Does your paper address subitem X26-iii

Copy and paste relevant sections from the manuscript (include quotes in quotation marks "like this" to indicate direct quotes from your manuscript), or elaborate on this item by providing additional information not in the ms, or briefly explain why the item is not applicable/relevant for your study

התשובה שלך

RESULTS

13a) For each group, the numbers of participants who were randomly assigned, received intended treatment, and were analysed for the primary outcome

NPT: The number of care providers or centers performing the intervention in each group and the number of patients treated by each care provider in each center

* ?Does your paper address CONSORT subitem 13a

Copy and paste relevant sections from the manuscript (include quotes in quotation marks "like this" to indicate direct quotes from your manuscript), or elaborate on this item by providing additional information not in the ms, or briefly explain why the item is not applicable/relevant for your study

Item 13a is applicable as it

13b) For each group, losses and exclusions after randomisation, together with reasons

* Does your paper address CONSORT subitem 13b? (NOTE: Preferably, this is shown in a CONSORT flow diagram)

Copy and paste relevant sections from the manuscript (include quotes in quotation marks "like this" to indicate direct quotes from your manuscript), or elaborate on this item by providing additional information not in the ms, or briefly explain why the item is not applicable/relevant for your study

Item 13b is not applicable as there were no losses or exclusions after randomization

13b-i) Attrition diagram

Strongly recommended: An attrition diagram (e.g., proportion of participants still logging in or using the intervention/comparator in each group plotted over time, similar to a survival .curve) or other figures or tables demonstrating usage/dose/engagement

	5	4	3	2	1	
essential	○	○	○	○	○	subitem not at all important

?Does your paper address subitem 13b-i

Copy and paste relevant sections from the manuscript or cite the figure number if applicable (include quotes in quotation marks "like this" to indicate direct quotes from your manuscript), or elaborate on this item by providing additional information not in the ms, or briefly explain why the item is not applicable/relevant for your study

התשובה שלך

14a) Dates defining the periods of recruitment and follow-up

?Does your paper address CONSORT subitem 14a *

Copy and paste relevant sections from the manuscript (include quotes in quotation marks "like this" to indicate direct quotes from your manuscript), or elaborate on this item by providing additional information not in the ms, or briefly explain why the item is not applicable/relevant for your study

Item 14 a is applicable as the follow- up periods were defined in the text.

14a-i) Indicate if critical “secular events” fell into the study period

Indicate if critical “secular events” fell into the study period, e.g., significant changes in Internet resources available or “changes in computer hardware or Internet delivery resources”

	5	4	3	2	1	
essential	○	○	○	○	○	subitem not at all important

?Does your paper address subitem 14a-i

Copy and paste relevant sections from the manuscript (include quotes in quotation marks "like this" to indicate direct quotes from your manuscript), or elaborate on this item by providing additional information not in the ms, or briefly explain why the item is not applicable/relevant for your study

התשובה שלך

14b) Why the trial ended or was stopped (early)

* ?Does your paper address CONSORT subitem 14b

Copy and paste relevant sections from the manuscript (include quotes in quotation marks "like this" to indicate direct quotes from your manuscript), or elaborate on this item by providing additional information not in the ms, or briefly explain why the item is not applicable/relevant for your study

Item 14b is not applicable as the study was not ended earlier

A table showing baseline demographic and clinical characteristics for each (15 group

NPT: When applicable, a description of care providers (case volume, qualification, expertise, etc.) and centers (volume) in each group

* ?Does your paper address CONSORT subitem 15

Copy and paste relevant sections from the manuscript (include quotes in quotation marks "like this" to indicate direct quotes from your manuscript), or elaborate on this item by providing additional information not in the ms, or briefly explain why the item is not applicable/relevant for your study

Item 15 is applicable, a table describing the demographic characteristics for each group was added

i) Report demographics associated with digital divide issues-15

In ehealth trials it is particularly important to report demographics associated with digital divide issues, such as age, education, gender, social-economic status, .computer/Internet/ehealth literacy of the participants, if known

	5	4	3	2	1	
essential	○	○	○	○	○	subitem not at all important

* ?Does your paper address subitem 15-i

Copy and paste relevant sections from the manuscript (include quotes in quotation marks "like this" to indicate direct quotes from your manuscript), or elaborate on this item by providing additional information not in the ms, or briefly explain why the item is not applicable/relevant for your study

Item 15i is applicable as the demographics were reported as known

For each group, number of participants (denominator) included in each analysis (16 and whether the analysis was by original assigned groups

i) Report multiple “denominators” and provide definitions-16

Report multiple “denominators” and provide definitions: Report N’s (and effect sizes) “across a range of study participation [and use] thresholds” [1], e.g., N exposed, N consented, N used more than x times, N used more than y weeks, N participants “used” the intervention/comparator at specific pre-defined time points of interest (in absolute and .relative numbers per group). Always clearly define “use” of the intervention

	5	4	3	2	1	
essential	○	○	○	○	○	subitem not at all important

* ?Does your paper address subitem 16-i

Copy and paste relevant sections from the manuscript (include quotes in quotation marks "like this" to indicate direct quotes from your manuscript), or elaborate on this item by providing additional information not in the ms, or briefly explain why the item is not applicable/relevant for your study

Item 16i is applicable as the denominators were described in the text and in figure 3.

ii) Primary analysis should be intent-to-treat-16

Primary analysis should be intent-to-treat, secondary analyses could include comparing only “users”, with the appropriate caveats that this is no longer a randomized sample (see .18-i)

	5	4	3	2	1	
essential	○	○	○	○	○	subitem not at all important

?Does your paper address subitem 16-ii

Copy and paste relevant sections from the manuscript (include quotes in quotation marks "like this" to indicate direct quotes from your manuscript), or elaborate on this item by providing additional information not in the ms, or briefly explain why the item is not applicable/relevant for your study

התשובה שלך

17a) For each primary and secondary outcome, results for each group, and the estimated effect size and its precision (such as 95% confidence interval)

* ?Does your paper address CONSORT subitem 17a

Copy and paste relevant sections from the manuscript (include quotes in quotation marks "like this" to indicate direct quotes from your manuscript), or elaborate on this item by providing additional information not in the ms, or briefly explain why the item is not applicable/relevant for your study

Item 17a is applicable, the effect size is added in the text.

17a-i) Presentation of process outcomes such as metrics of use and intensity of use

In addition to primary/secondary (clinical) outcomes, the presentation of process outcomes such as metrics of use and intensity of use (dose, exposure) and their operational definitions is critical. This does not only refer to metrics of attrition (13-b) (often a binary variable), but also to more continuous exposure metrics such as “average session length”. These must be accompanied by a technical description how a metric like a .“session” is defined (e.g., timeout after idle time) [1] (report under item 6a)

	5	4	3	2	1	
essential	○	○	○	○	○	subitem not at all important

?Does your paper address subitem 17a-i

Copy and paste relevant sections from the manuscript (include quotes in quotation marks "like this" to indicate direct quotes from your manuscript), or elaborate on this item by providing additional information not in the ms, or briefly explain why the item is not applicable/relevant for your study

התשובה שלך

17b) For binary outcomes, presentation of both absolute and relative effect sizes is recommended

* ?Does your paper address CONSORT subitem 17b

Copy and paste relevant sections from the manuscript (include quotes in quotation marks "like this" to indicate direct quotes from your manuscript), or elaborate on this item by providing additional information not in the ms, or briefly explain why the item is not applicable/relevant for your study

Item 17 b Cramer V test was applied for measuring the effect size of correlation between categorical fields and Cohen d for measuring the effect size of the differences between 2 group means

Results of any other analyses performed, including subgroup analyses and (18 adjusted analyses, distinguishing pre-specified from exploratory

?Does your paper address CONSORT subitem 18 *

Copy and paste relevant sections from the manuscript (include quotes in quotation marks "like this" to indicate direct quotes from your manuscript), or elaborate on this item by providing additional information not in the ms, or briefly explain why the item is not applicable/relevant for your study

Subitem 18 was applicable as the relationship between the awareness of the risk from influenza and vaccination rate only in group B using chi-square test was tested.

i) Subgroup analysis of comparing only users-18

A subgroup analysis of comparing only users is not uncommon in ehealth trials, but if done, it must be stressed that this is a self-selected sample and no longer an unbiased .sample from a randomized trial (see 16-iii)

	5	4	3	2	1	
essential	○	○	○	○	○	subitem not at all important

?Does your paper address subitem 18-i

Copy and paste relevant sections from the manuscript (include quotes in quotation marks "like this" to indicate direct quotes from your manuscript), or elaborate on this item by providing additional information not in the ms, or briefly explain why the item is not applicable/relevant for your study

התשובה שלך

All important harms or unintended effects in each group (19
(for specific guidance see CONSORT for harms)

* ?Does your paper address CONSORT subitem 19

Copy and paste relevant sections from the manuscript (include quotes in quotation marks "like this" to indicate direct quotes from your manuscript), or elaborate on this item by providing additional information not in the ms, or briefly explain why the item is not applicable/relevant for your study

Item 19 is not applicable as no harms or unintended effects were applied, The study analyzed retrospectively the outcomes.

i) Include privacy breaches, technical problems-19

Include privacy breaches, technical problems. This does not only include physical "harm" to participants, but also incidents such as perceived or real privacy breaches [1], technical problems, and other unexpected/unintended incidents. "Unintended effects" also includes .unintended positive effects [2]

	5	4	3	2	1	
essential	○	○	○	○	○	subitem not at all important

?Does your paper address subitem 19-i

Copy and paste relevant sections from the manuscript (include quotes in quotation marks "like this" to indicate direct quotes from your manuscript), or elaborate on this item by providing additional information not in the ms, or briefly explain why the item is not applicable/relevant for your study

התשובה שלך

ii) Include qualitative feedback from participants or observations from-19 staff/researchers

Include qualitative feedback from participants or observations from staff/researchers, if available, on strengths and shortcomings of the application, especially if they point to unintended/unexpected effects or uses. This includes (if available) reasons for why people .did or did not use the application as intended by the developers

	5	4	3	2	1	
essential	○	○	○	○	○	subitem not at all important

?Does your paper address subitem 19-ii

Copy and paste relevant sections from the manuscript (include quotes in quotation marks "like this" to indicate direct quotes from your manuscript), or elaborate on this item by providing additional information not in the ms, or briefly explain why the item is not applicable/relevant for your study

התשובה שלך

DISCUSSION

Interpretation consistent with results, balancing benefits and harms, and (22 considering other relevant evidence

NPT: In addition, take into account the choice of the comparator, lack of or partial blinding, and unequal expertise of care providers or centers in each group

i) Restate study questions and summarize the answers suggested by the data,-22 starting with primary outcomes and process outcomes (use)

Restate study questions and summarize the answers suggested by the data, starting with .primary outcomes and process outcomes (use)

	5	4	3	2	1	
essential	○	○	○	○	○	subitem not at all important

?Does your paper address subitem 22-i *

Copy and paste relevant sections from the manuscript (include quotes in quotation marks "like this" to indicate direct quotes from your manuscript), or elaborate on this item by providing additional information not in the ms, or briefly explain why the item is not applicable/relevant for your study

Item 22-i is addressed, the discussion starts with description of the principle findings.

ii) Highlight unanswered new questions, suggest future research-22

.Highlight unanswered new questions, suggest future research

	5	4	3	2	1	
essential	○	○	○	○	○	subitem not at all important

?Does your paper address subitem 22-ii

Copy and paste relevant sections from the manuscript (include quotes in quotation marks "like this" to indicate direct quotes from your manuscript), or elaborate on this item by providing additional information not in the ms, or briefly explain why the item is not applicable/relevant for your study

התשובה שלך

Trial limitations, addressing sources of potential bias, imprecision, and, if (20
relevant, multiplicity of analyses

i) Typical limitations in ehealth trials-20

Typical limitations in ehealth trials: Participants in ehealth trials are rarely blinded. Ehealth trials often look at a multiplicity of outcomes, increasing risk for a Type I error. Discuss biases due to non-use of the intervention/usability issues, biases through informed .consent procedures, unexpected events

	5	4	3	2	1	
essential	○	○	○	○	○	subitem not at all important

* ?Does your paper address subitem 20-i

Copy and paste relevant sections from the manuscript (include quotes in quotation marks "like this" to indicate direct quotes from your manuscript), or elaborate on this item by providing additional information not in the ms, or briefly explain why the item is not applicable/relevant for your study

Subitem 20i is addressed, the limitations of the study are added in the discussion

Generalisability (external validity, applicability) of the trial findings (21

NPT: External validity of the trial findings according to the intervention, comparators, patients, and care providers or centers involved in the trial

i) Generalizability to other populations-21

Generalizability to other populations: In particular, discuss generalizability to a general Internet population, outside of a RCT setting, and general patient population, including applicability of the study results for other organizations

	5	4	3	2	1	
essential	○	○	○	○	○	subitem not at all important

?Does your paper address subitem 21-i

Copy and paste relevant sections from the manuscript (include quotes in quotation marks "like this" to indicate direct quotes from your manuscript), or elaborate on this item by providing additional information not in the ms, or briefly explain why the item is not applicable/relevant for your study

התשובה שלך

ii) Discuss if there were elements in the RCT that would be different in a routine-21 application setting

Discuss if there were elements in the RCT that would be different in a routine application setting (e.g., prompts/reminders, more human involvement, training sessions or other co-interventions) and what impact the omission of these elements could have on use, .adoption, or outcomes if the intervention is applied outside of a RCT setting

	5	4	3	2	1	
essential	○	○	○	○	○	subitem not at all important

?Does your paper address subitem 21-ii

Copy and paste relevant sections from the manuscript (include quotes in quotation marks "like this" to indicate direct quotes from your manuscript), or elaborate on this item by providing additional information not in the ms, or briefly explain why the item is not applicable/relevant for your study

התשובה שלך

OTHER INFORMATION

Registration number and name of trial registry (23

* ?Does your paper address CONSORT subitem 23

Copy and paste relevant sections from the manuscript (include quotes in quotation marks "like this" to indicate direct quotes from your manuscript), or elaborate on this item by providing additional information not in the ms, or briefly explain why the item is not applicable/relevant for your study

Subitem 23 is addressed the study is registered in Clinicaltrials.gov

Where the full trial protocol can be accessed, if available (24

* ?Does your paper address CONSORT subitem 24

Cite a Multimedia Appendix, other reference, or copy and paste relevant sections from the manuscript (include quotes in quotation marks "like this" to indicate direct quotes from your manuscript), or elaborate on this item by providing additional information not in the ms, or briefly explain why the item is not applicable/relevant for your study

Subitem 24 is addressed as a part of the registration, study plan was submitted.
ClinicalTrials.gov NCT06840236

Sources of funding and other support (such as supply of drugs), role of funders (25

* ?Does your paper address CONSORT subitem 25

Copy and paste relevant sections from the manuscript (include quotes in quotation marks "like this" to indicate direct quotes from your manuscript), or elaborate on this item by providing additional information not in the ms, or briefly explain why the item is not applicable/relevant for your study

Item 25 is addressed, Dario Health funded the study as mentioned in the text

X27) Conflicts of Interest (not a CONSORT item)

X27-i) State the relation of the study team towards the system being evaluated

In addition to the usual declaration of interests (financial or otherwise), also state the relation of the study team towards the system being evaluated, i.e., state if the authors/evaluators are distinct from or identical with the developers/sponsors of the .intervention

	5	4	3	2	1	
essential	○	○	○	○	○	subitem not at all important

?Does your paper address subitem X27-i

Copy and paste relevant sections from the manuscript (include quotes in quotation marks "like this" to indicate direct quotes from your manuscript), or elaborate on this item by providing additional information not in the ms, or briefly explain why the item is not applicable/relevant for your study

התשובה שלך

About the CONSORT EHEALTH checklist

?As a result of using this checklist, did you make changes in your manuscript *

- yes, major changes
- yes, minor changes
- no (selected)

What were the most important changes you made as a result of using this ?checklist

התשובה שלך

* How much time did you spend on going through the checklist INCLUDING making changes in your manuscript

I spent approximately 3 hours

* ?As a result of using this checklist, do you think your manuscript has improved

- ○ yes
- ◉ no
- ○ :אחר

?Would you like to become involved in the CONSORT EHEALTH group

This would involve for example becoming involved in participating in a workshop and writing an "Explanation and Elaboration" document

- ○ yes
- ○ no
- ○ :אחר

Any other comments or questions on CONSORT EHEALTH

התשובה שלך

STOP - Save this form as PDF before you click submit

To generate a record that you filled in this form, we recommend to generate a PDF of this .page (on a Mac, simply select "print" and then select "print as PDF") before you submit it

When you submit your (revised) paper to JMIR, please upload the PDF as supplementary .file

Don't worry if some text in the textboxes is cut off, as we still have the complete !information in our database. Thank you

! Final step: Click submit

!Click submit so we have your answers in our database

שליחה ניקוי הטופס

אין לשלוח סיסמאות באמצעות Google Forms.

תוכן זה לא נוצר ולא נתמך על ידי Google. - תנאים והגבלות - למדיניות הפרטיות

הפריט הזה נראה חשוד? דוח

Google Forms
